# Porcine influenza mAbs to H3, H5, and H7 hemagglutinins recognize H3 egg adapted site and target the HA stem

**DOI:** 10.1093/discim/kyag006

**Published:** 2026-03-02

**Authors:** Tiphaine Cayol, Sonia Villanueva-Hernández, Emily Briggs, Charlotte May, Adam McNee, Ashutosh Vats, Bharti Mittal, Jean-Remy Sadeyen, Munir Iqbal, Danish Munir, Marie Di Placido, John A Hammond, Alain Townsend, Pramila Rijal, Basudev Paudyal, Elma Tchilian

**Affiliations:** Host Response, The Pirbright Institute, Woking, UK; Host Response, The Pirbright Institute, Woking, UK; IrsiCaixa, Badalona, Barcelona, Spain; Host Response, The Pirbright Institute, Woking, UK; Host Response, The Pirbright Institute, Woking, UK; Host Response, The Pirbright Institute, Woking, UK; Host Response, The Pirbright Institute, Woking, UK; Host Response, The Pirbright Institute, Woking, UK; Host Response, The Pirbright Institute, Woking, UK; Host Response, The Pirbright Institute, Woking, UK; Host Response, The Pirbright Institute, Woking, UK; Host Response, The Pirbright Institute, Woking, UK; Host Response, The Pirbright Institute, Woking, UK; Chinese Academy of Medical Science Oxford Institute, Nuffield Department of Medicine, University of Oxford, Oxford, UK; MRC Translational Immune Discovery Unit, Weatherall Institute of Molecular Medicine, Radcliffe Department of Medicine, University of Oxford, Oxford, UK; Chinese Academy of Medical Science Oxford Institute, Nuffield Department of Medicine, University of Oxford, Oxford, UK; MRC Translational Immune Discovery Unit, Weatherall Institute of Molecular Medicine, Radcliffe Department of Medicine, University of Oxford, Oxford, UK; Host Response, The Pirbright Institute, Woking, UK; Host Response, The Pirbright Institute, Woking, UK

**Keywords:** pig monoclonal antibodies, H3, H5, H7, H3 egg adaptation H3N2, stem influenza antibodies

## Abstract

**Introduction:**

Monoclonal antibodies (mAbs) are critical tools for elucidating viral evolution, informing vaccine design, and developing antiviral therapeutics. Large-animal models, such as the pig, that closely mirror human immune responses are essential for understanding influenza immunity.

**Methods:**

Pigs were either infected or sequentially immunized with influenza viruses and monoclonal antibodies directed against H3, H5, and H7 influenza virus haemagglutinins were isolated. Antibody specificity, breadth, epitope targeting (head versus stem), neutralizing capacity, and Fc-mediated activity were assessed across influenza subtypes.

**Results:**

Pigs generated both strain-specific and broadly reactive mAbs targeting haemagglutinin head and stem epitopes. An H3-specific mAb (H3–57) selectively recognized the egg-adapted L194P mutation associated with reduced human vaccine effectiveness. H5 and H7 immunization induced neutralizing antibodies, including cross-group stem mAbs reactive with H1, H3, and H5 haemagglutinins. Fc-mediated activity correlated with antibody binding strength rather than epitope location.

**Conclusions:**

These findings demonstrate that pigs mount antibody responses closely resembling those observed in humans, including recognition of conserved stem epitopes and adaptive head mutations. Porcine mAbs represent powerful new tools for dissecting influenza immunity, guiding vaccine design, and enhancing pandemic preparedness using a physiologically relevant large-animal model.

## Graphical Abstract

**Graphical Abstract:**
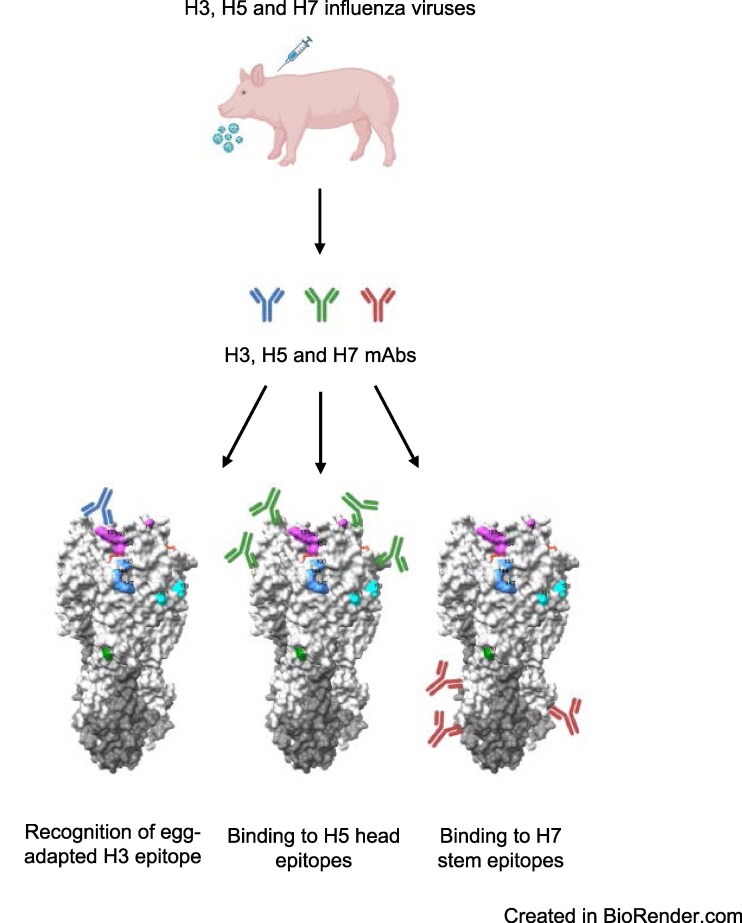

## Introduction

Pigs are natural hosts for influenza A viruses, with H1N1 and H3N2 subtypes co-circulating in both pigs and humans [1–3]. Frequent bidirectional transmission between the two species contributes to viral evolution and can give rise to novel pandemic strains [4–7]. Unlike small laboratory animals, pigs more closely resemble humans anatomically, genetically, physiologically, and immunologically [8–10]. They develop similar clinical signs and lung pathology following influenza infection, and the lobar and bronchial anatomy, as well as the histological structure of their lungs, closely mirrors that of humans [1, 11]. Pigs also exhibit a comparable distribution of sialic acid receptors to humans in the respiratory tract, which determines viral tropism [12, 13]. Their longer lifespans, unrestricted access to the respiratory tract and large body size make them an excellent model that better reflects human disease for studying influenza virus infection dynamics, immunity, and vaccine responses. Furthermore, an expanding toolkit—including inbred pig strains, tetramers, antibodies against immune markers and cytokines, and single-cell RNA sequencing—enables detailed characterization of porcine immune responses [14, 15]. Taken together these features make pigs a valuable translational model for influenza research.

Monoclonal antibodies (mAbs) have been extensively used to define the antigenic structure of the influenza haemagglutinin (HA) glycoprotein and elucidate mechanisms of viral immune escape. Studies in mice and humans have defined three broad categories of anti-HA antibodies. The first, consists of highly potent neutralizing mAbs directed to the immunodominant HA head domain; these antibodies are typically strain-, subtype-, or clade-specific [16, 17]. The second group also targets the HA head but exhibits broader reactivity, with cross-neutralization within a given subtype. The third group is characterized by broadly neutralizing mAbs that cross-react across multiple HA subtypes and are largely directed against the HA stem region [18, 19]. These mAbs have provided important insights into influenza antigenicity and led to development of antibody-based therapeutics and novel immunization strategies.

We have previously shown that pigs infected with the H1N1 pandemic 2009 virus generated mAbs that recognize the same antigenic sites as human antibodies [20]. Interestingly, one of these epitopes was not detected by ferret antisera, which are routinely used in global influenza surveillance and vaccine strain selection. In the present study, we extended this work by generating mAbs against seasonal H3N2 virus and characterized their functional properties and binding epitopes. We also sequentially immunized pigs with the human quadrivalent inactivated vaccine (QIV), followed by single cycle replication influenza pseudotyped viruses H5- and H7-S-FLUs [21], to induce responses to H5 and H7 HAs and determine whether anti-stem mAbs could be elicited. The resulting porcine mAbs were analysed for their binding specificity and functional activity. Here their relevance for better understanding virus evolution and their potential for developing novel therapeutic strategies is discussed.

## Results

### Generation and characterization of porcine H3-specific mAbs

**Figure 1:**
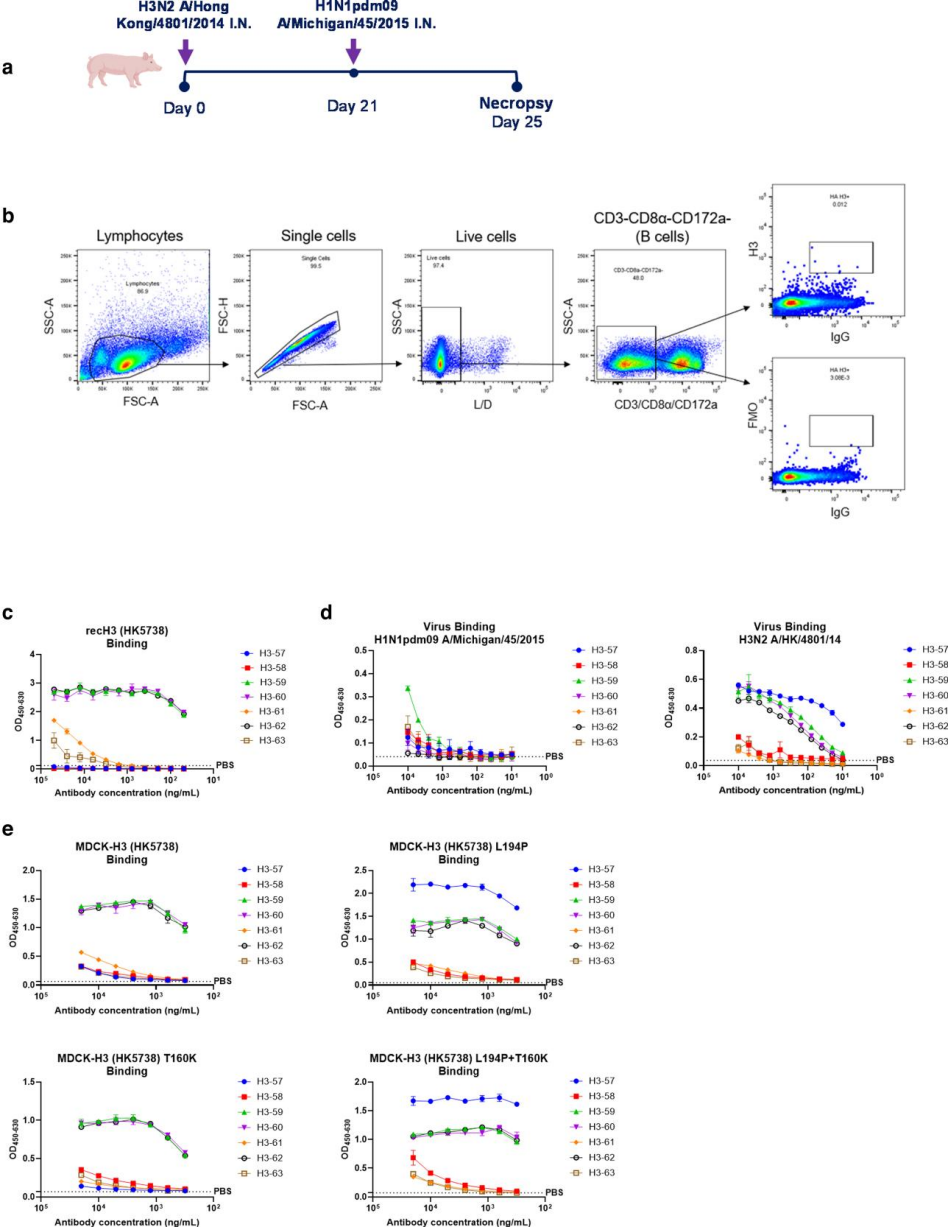
Generation and characterization of H3-specific porcine mAbs. (a) Experimental design of animal H3N2/pH1N1 challenge study. Six 5-weeks old pigs were infected with H3N2 A/Hong Kong/4801/2014. Three weeks later all the pigs were challenged with H1N1pdm09 A/Michigan/45/2015. Four-days post challenge with H1N1, all the pigs were culled, and tissues were collected. (b**)** Gating strategy for sorting single H3-specific IgG positive B cells. H3-specific B cells were sorted in a 96-well plate using recH3 protein from A/Hong Kong/5738/2014 (HK5738). (c) mAbs binding to rec H3 (HK5738) or (d**)** H1N1pdm09 A/Michigan/45/2015 or H3N2 A/Hong Kong/4801/2014 (e) Binding with MDCK-H3 (HK5738) and its HA mutants. All assays were performed in at least two independent experiments, each conducted in duplicate. Error bars represent the standard deviation.

H3-specific mAbs were isolated from pigs infected intranasally first with H3N2 (A/Hong Kong/4801/2014; HK4801), followed by H1N1 (A/Michigan/45/2015; Mich45) 3 weeks later (Fig. 1a). Antigen-specific B cells were isolated from tracheobronchial lymph nodes (TBLN) using recombinant H3 protein (rec H3) from A/Hong Kong/5738/2014 (HK5738) (Fig. 1b, Supplementary Figure S1). Seven mAb pairs were generated, consisting of two kappa and five lambda light chains (Table 1).

**Table 1.** H3-specific porcine mAbs characterization.

	Gene structure						Binding affinity								neutralization (ng/ml)			Fc-mediated functions	
H3-specific mAb	Light chain V family	VH gene family			CDRH3 length (aa)	No. of Cysteines in CDRH3	recH3 HK5738	MDCK-H3	MDCK-H3	MDCK-H3	MDCK-H3	MDCK-H1 Eng195	MDCK-H5 VN1203	MDCK-H7 Anh1	H3 A/Hong Kong/5738/2014 S-FLU	H3N2 A/Hong Kong/4801/2014	H1N1pdm09 A/Michigan/45/2015	CDCC (% Lysis)	NK-degranulation
								HK5738	HK5738	HK5738	HK5738								
									L194P	T160K	L194P + T160K								
H3-57	IGKV2	IGHV1S2*01	IGHD1*02	IGHJ5*01	20	2	—	+	+++	—	+++	nd	nd	nd	—	2.938	—	++	nd
H3-58	IGKV2	IGHV1-12*01	IGHD2*01	IGHJ5*01	14	0	—	+	+	+	+	+	+	+	—	—	—	nd	nd
H3-59	IGLV8	IGHV1-8*01	IGHD2*01	IGHJ5*01	19	2	+++	+++	+++	+++	+++	nd	nd	nd	202.9	102.2	—	++	+
H3-60	IGLV8	IGHV1-8*01	IGHD2*01	IGHJ5*01	19	2	+++	+++	+++	+++	+++	nd	nd	nd	200.4	187.5	—	++	++
H3-61	IGLV8	IGHV1-15*01	IGHD1*01,IGHD1*02	IGHJ5*01	17	1	+	+	+	+	+	—	—	—	—	—	—	+	+
H3-62	IGLV8	IGHV1-8*01	IGHD2*01	IGHJ5*01	19	2	+++	+++	+++	+++	+++	nd	nd	nd	84.54	167.3	—	++	+
H3-63	IGLV3	IGHV1-15*01	IGHD1*02	IGHJ5*01	20	1	+	+	+	+	+	++	++	++	—	—	—	+	+

For binding affinity to recH3 (HK5738): –, no detectable binding at starting concentration of 50 000 ng/ml; +, weak binding, OD_450–630_ < 2.0; +++, strong binding, OD_450–630_ ≥ 2.0. For binding affinity to MDCK-H3: —, no detectable binding at starting concentration 20 000 ng/ml; +, weak binding, OD_450–630_ < 0.75; +++, strong binding, OD_450–630_ ≥ 0.75. For binding affinity to MDCK-H1 (Eng195), MDCK-H5 (VN1203) and MDCK-H7 (Anh1): —, no detectable binding at starting concentration 50 000 ng/ml; +, weak binding, OD_450–630_ < 0.5; ++, intermediate binding, OD_450–630_ ≥ 0.5 and < 1.0; +++, strong binding, OD_450–630_ ≥ 1.0. For neutralizing activity, —, non-neutralizing; and for neutralizers, the concentration at which 50% of the viruses are inhibited, defined as the IC_50_, is indicated. For CDCC: —, no detectable lysis; +, detectable lysis (<30%) at starting mAb concentration of 40 ug/ml; ++, detectable lysis (>30%) at starting mAb concentration of 10 ug/ml. For ADCC: +, <30% CD107a expressing NK cells; ++, ≥30% CD107a expressing NK cells. nd: not determined.

**Figure 2:**
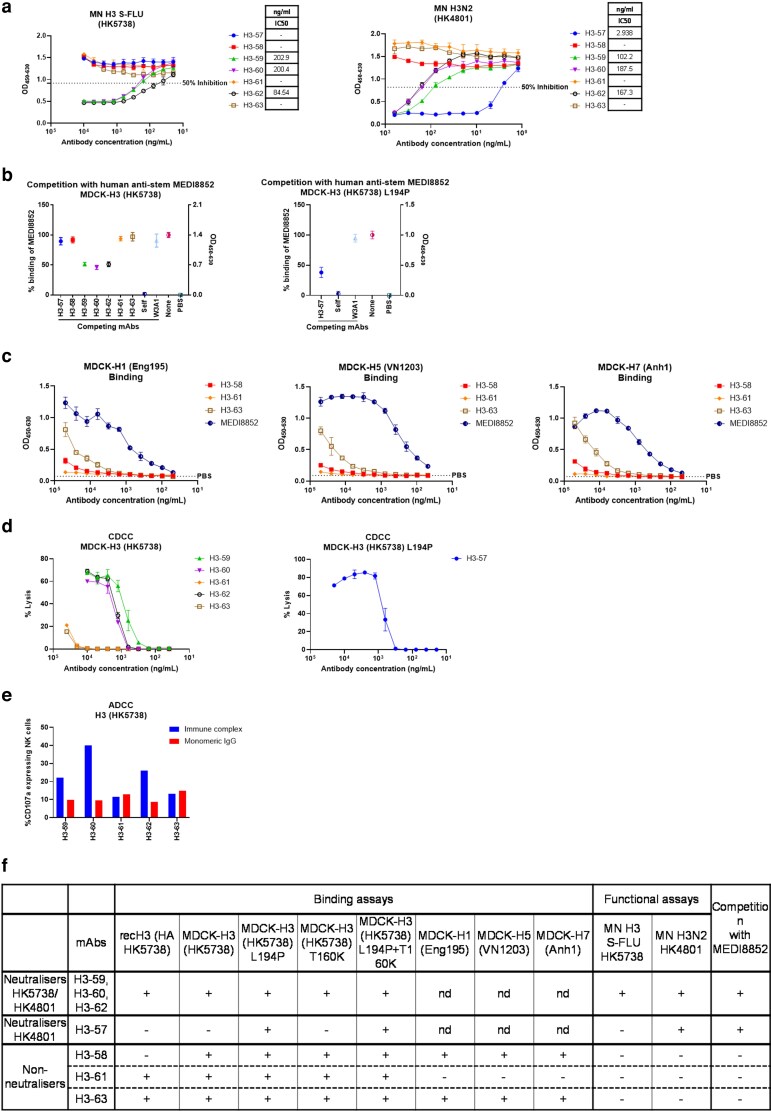
Generation and characterization of H3-specific porcine mAbs. (a) Concentration of individual mAb giving 50% neutralization (IC50) against H3 S-FLU A/HK/5738/14 or H3N2 A/HK/4801/14. (b) Competition with MEDI8852 for binding to MDCK-H3 (HK5738) and MDCK-H3 (HK5738) L194P cells. (c) mAbs binding to MDCK-H1 (Eng195), MDCK-H5 (VN1203), and MDCK-H7 (Anh1). (d**)** Complement-dependent cell cytotoxicity (CDCC) on MDCK-H3 (HK5738) and MDCK-H3 (HK5738) L194P cells incubated with the H3-mAbs in presence of rabbit complement. (e) ADCC was quantified by percentage of NK cells expressing CD107a. (f) Summary table of H3 mAbs. nd: not determined. Error bars represent the standard deviation.

The seven H3-specific mAbs (H3-57 to H3-63) were recombinantly expressed and characterized for their binding, neutralization, haemagglutination inhibition (HAI), and Fc-effector functions (Table 1). The binding affinity was categorized based on the absorbance value recorded at different concentrations (Table 1). Three mAbs (H3-59, H3-60, and H3-62) bound strongly to the rec H3 (HK5738) protein used for sorting, while H3-61 and H3-63, showed weaker binding (Fig. 1c). Interestingly, mAb H3-57, did not bind to rec H3 (HK5738) (Fig. 1c). It bound to the challenge virus HK4801 but not to MDCK cells expressing H3 (HK5738) (Figs. 1d and e). To determine whether this binding difference was related to the egg-adaptive substitutions in the egg-derived challenge virus HK4801, we tested the binding of mAb H3-57 to MDCK cells expressing ΔHK5738 HA (T160K, L194P and double mutant T160K + L194P, key egg-adaptive changes in H3N2 vaccines within the antigenic site B). T160K led to loss of a glycosylation site at HA position 158 and L194P also altered the antigenic site B. These mutations were responsible for reduced vaccine effectiveness in 2016–2018 [22, 23]. MAb H3-57 bound only to the HA with proline at position 194 (Fig. 1e). Furthermore, H3-57 neutralized the egg-grown HK4801 virus with IC50 values of 2.9 ng/ml (Fig. 2a), suggesting that pigs can make antibodies targeting site B that distinguish between egg-derived and circulating viruses.

The binding hierarchy to MDCK-H3 (HK5738) cells was very similar to the binding to rec H3 (HK5738) with mAbs H3-59, H3-60, and H3-62 exhibiting the greatest binding (Fig. 1e). The binding profiles of the H3-specific mAbs corresponded with their neutralization activities. Only the strong binders, H3-59, H3-60, and H3-62, effectively neutralized H3 S-FLU (HK5738) with IC50 values of 202 ng/ml, 200 ng/ml, and 84 ng/ml, respectively (Fig. 2a). None of the H3-specific mAbs neutralized the H1N1 (Mich45) challenge virus (Supplementary FigureS2)

To identify the site recognized by neutralizing H3-specific mAbs, neutralization of mAbs H3-59, H3-60 and H3-62 was tested to eleven HK5738 S-FLU viruses carrying single residue substitution within the known antigenic sites, namely Site A (T135K, S144N/K, R142G), Site B (K189E, T160K, F193S, L194P), Site C (E50K), and Site D (N121K, N171K) (Supplementary Figure S3). These substitutions abrogate neutralization by site-matched human mAbs and are established drivers of H3 antigenic evolution [24]. All three mAbs neutralized all eleven variant viruses at a single concentration of 50 μg/ml. (Supplementary Table S1), suggesting that these three neutralizing mAbs detect sites other than known canonical H3 head antigenic sites. Because contemporary H3N2 viruses exhibit reduced haemagglutination of chicken red blood cells, we were unable to evaluate these mAbs using the standard HAI [25].

To assess the potential of the H3-specifc mAbs to target stem epitopes, the inhibition of binding of the human anti-stem mAb MEDI8852 [26] to MDCK-H3 (H5738) was analysed. However, although mAbs H3-59, H3-60, and H3-62 inhibited MEDI8852 binding, the inhibition did not exceed 50%, suggesting that the effect was more likely due to steric hindrance than to genuine stem-directed activity (Fig. 2b). Interestingly, the non-neutralizing mAb H3-63 cross-reacted with H1, H5, and H7 expressed on MDCK (MDCK-H1 (Eng195), MDCK-H3 (HK5738) and MDCK-H7 (Anh1)) cells (Fig. 2c), showing both group I and group II cross-reactivity typical of a stem-directed mAb. However, it did not compete for binding with the MEDI8852 (Fig. 2b), likely due to its weak binding affinity (Figs. 1c, 1e). The mAb competition results are difficult to interpret for low affinity antibodies, especially in the case of one-way blocking of a strong binding mAb.

The Fc-mediated effector functions of the porcine H3 mAbs, complement-dependent cytotoxicity (CDCC) and antibody-dependent cellular cytotoxicity (ADCC) were assessed. Strong and moderate binders were tested for CDCC using MDCK-H3 (HK5738) cells and rabbit sera as source of complement. To test CDCC activity of H3-57, which only binds to the L194P mutant H3, MDCK-H3 cells expressing L194P mutant H3 were used. The strong binders and neutralizers H3-59, H3-60, and H3-62 exhibited strong complement activation, whereas the weaker binders, H3-61 and H3-63, demonstrated limited complement activity at the highest mAb concentrations only (Fig. 2d). Consistent with these findings, the strong binders also induced robust NK cell degranulation as measured by CD107a expression, while the weak binders failed to elicit any NK cell response (Fig. 2e). There was no association between IGHV, IGKV, or IGLV gene usage or CDRH3 length with neutralization, binding, or Fc-mediated functions (Table 1).

In summary, three types of H3-specific mAbs were identified (Fig. 2f). The first group comprised strong H3 (HK5738) binders (H3-59, H3-60, and H3-62), which exhibited neutralizing activity and targeted epitopes different from the canonical H3 head antigenic sites. The second group (H3-58, H3-61, and H3-63) consisted of weak binders with no detectable neutralizing, and low CDCC activity. Their epitopes remain uncharacterized, although H3-63 potentially targets a stem epitope. The third group, represented by mAb H3-57, recognized antigenic site B with P194 binding dependency of HK4801, indicating that, similar to humans, pigs can generate antibodies targeting site B that distinguish between egg-derived and circulating H3N2 virus isolates.

### Generation and characterization of porcine H5-specific mAbs

**Figure 3:**
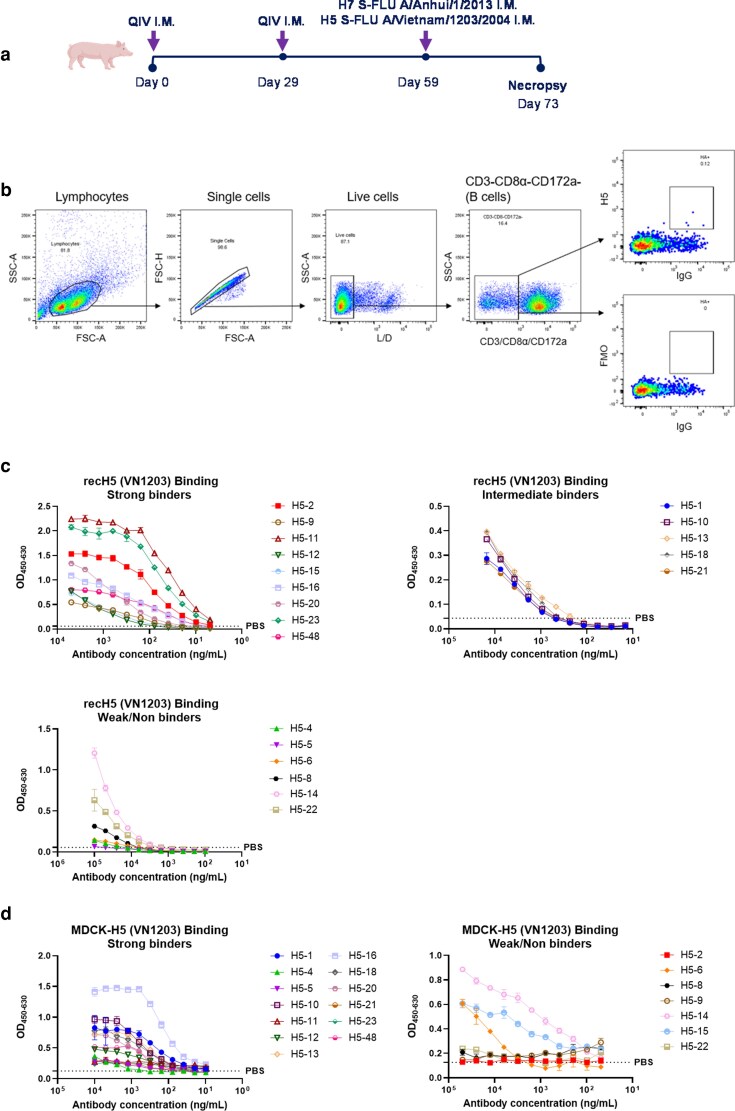
Generation and characterization of H5-specific porcine mAbs. (a) Experimental design of immunization study. Two 6-weeks old pigs were immunized intramuscularly with 2018/2019 QIV. Twenty-nine days later, the animals were boosted with QIV using the same dose and the route. Thirty days after the boost, animals were immunized with H5N1 S-FLU (H5: A/Vietnam/1203/2004, N1: A/PR/8/34) and H7N1 S-FLU (H7: A/Anhui/1/2013, N1: A/PR/8/34) in equal proportions. Five days post immunization with S-FLUs, pigs were culled, and tissues were harvested for sorting antigen-specific B cells. (b) Gating strategy to sort single H5-specific IgG positive B cells. H5-specific B cells were sorted in 96-well plate using recH5 protein from A/VN1203/04 (VN1203). (c) Binding to recH5 (VN1203) (d) and MDCK-H5 (VN1203) cells. All assays were performed in at least two independent experiments, each conducted in duplicate. Error bars represent the standard deviation.

Porcine H5-specific mAbs were generated from pigs sequentially immunized intramuscularly twice 29 days apart with the 2018/19 quadrivalent inactivated vaccine (QIV), followed by H5 S-FLU (H5 from A/Vietnam/1203/2004) (VN1203) and H7 S-FLU (H7 from A/Anhui/1/2013) (Anh1) intramuscular immunization 30 days later (Fig. 3a). S-FLU is a pseudotyped influenza virus lacking the HA signal sequence and therefore limited to a single cycle of replication [21]. It induces a strong HA antibody response when administered intramuscularly [21, 27]. Homologous rec H5 (VN1203) was used as a probe to isolate antigen-specific B cells from the retropharyngeal lymph nodes (RPLN) (Fig. 3b). The variable regions of the heavy and light chains were amplified using nested PCR, sequenced, and clustered. A total of 76 single cells were sorted, of which 57 were positive for both IgG heavy and light chains, resulting in a 75% recovery. From these clusters, 20 unique mAb sequences were selected and expressed recombinantly as IgG1. Among the selected mAbs, 10 had lambda light chains and 10 had kappa light chains, with heavy chains CDRH3 ranging from 8–27 amino acids length (Table 2).

**Table 2.** H5-specific porcine mAbs characterization.

	Gene structure						Binding affinity					neutralization (ng/ml)		Heamaglutination inhibition (ng/ml)	Fc-mediated functions	
H5-specific mAb	Light chain V family	VH gene family			CDRH3 length (aa)	No. of Cysteines in CDRH3	recH5 VN1203	MDCK-H5 VN1203	MDCK-H1 Eng195	MDCK-H3 HK5738	MDCK-H7 Anh1	H5 S-FLU A/VN/1203/2004	H5N1 S-FLU A/Texas/37/2024	H5 S-FLU A/VN/1203/2004	CDCC (% Lysis)	NK-degranulation
H5-1	IGLV8	IGHV1-10*01,IGHV1-4*01	IGHD2*01	IGHJ5*01	18	0	++	+++	nd	nd	nd	380	—	390.3	++	+
H5-2	IGLV8	IGHV1-10*01,IGHV1-4*01	IGHD1*01,IGHD1*02	IGHJ3*01	13	0	+++	—	—	—	—	—	—	—	nd	++
H5-4	IGLV3	IGHV1-10*01,IGHV1-4*01, IGHV1-4*02	IGHD1*02	IGHJ5*01	27	2	+	+++	nd	nd	nd	7000	—	9375	+	+
H5-5	IGLV8	IGHV1-14*01,IGHV1S2*01	IGHD1*01,IGHD1*02	IGHJ5*01	14	2	—	+++	—	—	—	—	—	—	+	+
H5-6	IGKV2	IGHV1-15*01	IGHD2*01	IGHJ5*01	17	1	+	+	nd	nd	nd	6880	—	—	+	+
H5-8	IGKV2	IGHV1S5*01	IGHD2*01	IGHJ5*01	17	1	+	—	—	—	—	—	—	—	nd	+
H5-9	IGLV8	IGHV1-10*01,IGHV1-4*01,IGHV1-4*02	IGHD3*01	IGHJ5*01	13	0	+++	—	—	—	—	—	—	—	nd	++
H5-10	IGLV8	IGHV1-10*01	IGHD1*01	IGHJ5*01	19	2	++	+++	nd	nd	nd	2020	—	3125	++	++
H5-11	IGKV1	IGHV1-4*02	IGHD1*01,IGHD1*02,IGHD4*01	IGHJ5*01	21	2	+++	+++	—	—	—	—	—	—	+	++
H5-12	IGLV8	IGHV1-15*01,IGHV1S2*01	None assigned	IGHJ5*01	8	0	+++	+++	—	—	—	—	—	—	+	++
H5-13	IGKV1	IGHV1-10*01,IGHV1-4*01, IGHV1-4*02	None assigned	IGHJ5*01	8	0	++	+++	—	—	—	—	—	—	++	+
H5-14	IGLV8	IGHV1-14*01	IGHD1*02	IGHJ5*01	11	0	+	+	—	—	—	—	—	—	+	+
H5-15	IGKV2	IGHV1S5*01	IGHD2*01	IGHJ5*01	15	1	+++	+	nd	nd	nd	3590	—	4687.5	+	+
H5-16	IGLV8	IGHV1-6*02	IGHD1*01,IGHD1*02,IGHD1*02,IGHD1*02	IGHJ5*01	21	2	+++	+++	nd	nd	nd	200	—	390.3	++	++
H5-18	IGKV1	IGHV1-10*01,IGHV1-4*01	IGHD1*01,IGHD1*02,IGHD1*02	IGHJ5*01	16	2	++	+++	nd	nd	nd	2340	—	18 750	++	+
H5-20	IGKV2	IGHV1-15*01	IGHD2*01	IGHJ5*01	17	1	+++	+++	nd	nd	nd	370	—	2344	++	++
H5-21	IGLV8	IGHV1-4*02	IGHD1*01,IGHD1*02,IGHD2*01,IGHD4*01	IGHJ5*01	20	2	++	+++	—	—	—	—	—	—	+	+
H5-22	IGKV2	IGHV1-10*01,IGHV1-4*01	IGHD3*01	IGHJ5*01	13	0	+	—	—	—	—	—	—	—	nd	+
H5- 23	IGKV2	IGHV1-15*01	IGHD2*01	IGHJ5*01	17	1	+++	+++	—	—	—	—	—	—	+	+
H5-48	IGKV2	IGHV1-15*01	IGHD1*01,IGHD1*02	IGHJ5*01,IGHJ5*02	17	1	+++	+++	—	—	—	—	—	—	+	++

For binding affinity to recH5 (VN1203) and MDCK-H5 (VN1203): -, no detectable binding; +, weak binding, ≥50 000 ng/ml; ++, intermediate binding, <50 000 ng/ml & > 10 000 ng/ml; +++, strong binding, ≤10 000 ng/ml. For binding affinity to MDCK-H1 (Eng195), MDCK-H3 (VN1203) and MDCK-H7 (Anh1): —, no detectable binding at starting concentration 50 000 ng/ml; +, weak binding, OD_450–630_ < 0.5; ++, intermediate binding, OD_450–630_ ≥ 0.5 & < 1.0; +++, strong binding, OD_450–630_ ≥ 1.0. For neutralizing activity, —, non-neutralizing; and for neutralizers, the concentration at which 50% of the viruses are inhibited, defined as the IC_50_, is indicated. Haemagglutination-inhibition activity is defined as the minimal concentration of the antibody required to prevent haemagglutination. For CDCC: —, no detectable lysis; +, detectable lysis at starting mAb concentration of 40 ug/ml; ++, detectable lysis at starting mAb concentration of 10 ug/ml. For ADCC: +, <30% CD107a expressing NK cells; ++, ≥ 30% CD107a expressing NK cells. nd: not determined.

H5-specific mAbs were initially characterized based on their binding activity to either rec H5 protein or MDCK expressing H5 (VN1203). Of the 20 H5-specific mAbs, 9 (H5-2, H5-9, H5-11, H5-12, H5-15, H5-16, H5-20, H5-23, and H5-48) demonstrated strong binding to rec H5 (VN1203). Five mAbs (H5-1, H5-10, H5-13, H5-18, and H5-21) showed intermediate binding, and six (H5-4, H5-5, H5-6, H5-8, H5-14, and H5-22) exhibited weak or no binding (Fig. 3c). Strength of binding was categorized based on the absorbance at different concentrations (Table 2). Binding analysis on MDCK-H5 (VN1203) cells demonstrated thirteen strong binders (H5-1, H5-4, H5-5, H5-10, H5-11, H5-12, H5-13, H5-16, H5-18, H5-20, H5-21, H5-23, and H5-48) and seven weak/non-binders (H5-2, H5-6, H5-8, H5-9, H5-14, H5-15, and H5-22) (Fig. 3d). Interestingly, mAbs such as H5-2 and H5-9 bound strongly to rec H5 but failed to bind to MDCK-H5 cells, likely due to conformational differences between native proteins expressed on the cell surface and recombinant HA proteins coated on the ELISA plate. Cell-surface HA may be partially cleaved into HA1/HA2, while recombinant proteins retain uncleaved HA0, affecting the stem/head interface epitopes.

**Figure 4:**
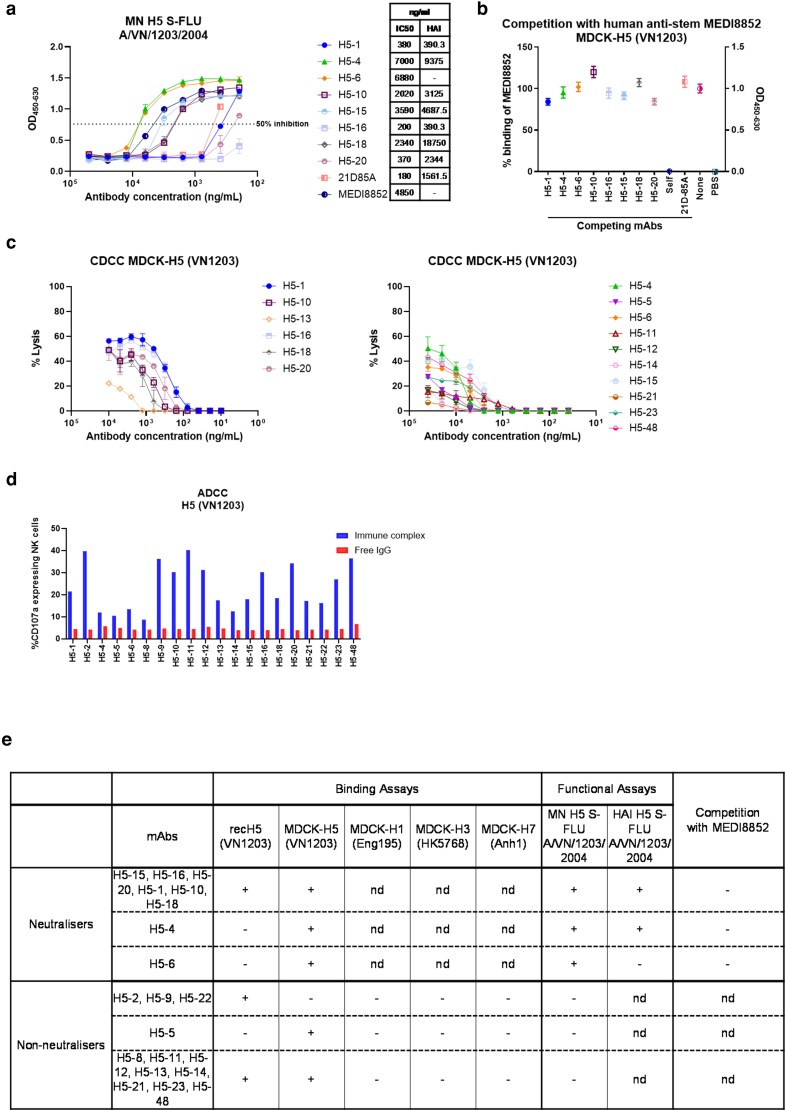
Functional activities of H5-specific porcine mAbs. (a) Concentration of individual mAbs giving 50% neutralization (IC50) against H5 S-FLU A/VN/1203/2004 and haemagglutination inhibition titres. (b) Competition with MEDI8852 for binding to MDCK-H5 (VN1203) cells. Human mAbs 21D-85A and MEDI8852 were used as controls. (c) Complement-dependent cell cytotoxicity (CDCC) on MDCK-H5 (VN1203) cells incubated with H5-mAbs in presence of rabbit complement. CDCC activity was measured by LDH release. (d) ADCC was quantified by percentage of NK cells expressing CD107a. (e) Summary table of H5 mAbs. nd: not determined. All assays were performed in at least two independent experiments, each conducted in duplicate. Error bars represent the standard deviation.

Next, we assessed the neutralizing activity of the H5 mAbs against VN1203 at an initial concentration of 50 000 ng/ml. Eight mAbs (H5-1, H5-4, H5-6, H5-10, H5-15, H5-16, H5-18, and H5-20) displayed neutralizing activity (Table 2). Further analysis of the concentration required for 50% inhibition of infection (IC50) showed values from 200 to 7000 ng/ml (Fig. 4a). There was no cross-neutralizing activity against the current circulating clade 2.3.4.4b dairy farm H5N1 strain (Table 2). All neutralizing mAbs also exhibited HAI, except for H5-6, suggesting that it is not a receptor binding site blocking antibody.

The inhibition of binding of the human anti-stem mAb MEDI8852 to MDCK-H5 (VN1203) by the H5-specifc mAbs was analysed. 21D85A, a human anti-H5 was used as a negative control/non-competitor. None of the H5-specific mAbs, including H5-6, competed with MEDI8852 (Fig. 4b). Furthermore, cross-reactive binding to other HAs (MDCK-H1, MCDK-H3, or MDCK-H7) was not detected (Supplementary Figure S4). Together with the HAI data, the lack of cross-reactivity and absence of stem competition suggest that these antibodies likely target head epitopes.

The Fc-mediated effector functions of the H5-specific mAbs were analysed. The strong binders (H5-1, H5-10, H5-13, H5-16, H5-18, and H5-20) exhibited robust complement activation, leading to up to 60% lysis of target cells at 10 μg/ml. The weak binders (H5-4, H5-5, H5-6, H5-11, H5-12, H5-14, H5-15, H5-21, H5-23, and H5-48) were able to activate complement at a higher concentration of 25 μg/ml, indicating an association between binding strength and complement activation (Fig. 4c). All H5 mAbs, induced NK cell degranulation (Fig. 4d). The most pronounced response was observed with H5-2, H5-9, H5-11, H5-20, and H5-48, where approximately 40% of NK cells expressed CD107a.

In summary, two groups of H5-specific mAbs were generated (Fig. 4e). The first group (H5-1, H5-4, H5-6, H5-10, H5-15, H5-16, H5-18, and H5-20) exhibited strong binding to MDCK-H5 VN1203, neutralizing activity, HAI activity (except H5-6), and Fc-mediated functions, most likely targeting head epitopes. In contrast, the majority of the second group of mAbs (H5-2, H5-5, H5-8, H5-9, H5-11, H5-12, H5-13, H5-14, H5-21, H5-22, H5-23, and H5-48) bound to rec H5 and MDCK-H5 cells but did not neutralize or inhibit haemagglutination, although some demonstrated CDCC activity.

### Generation and characterization of porcine H7-specific mAbs

**Figure 5:**
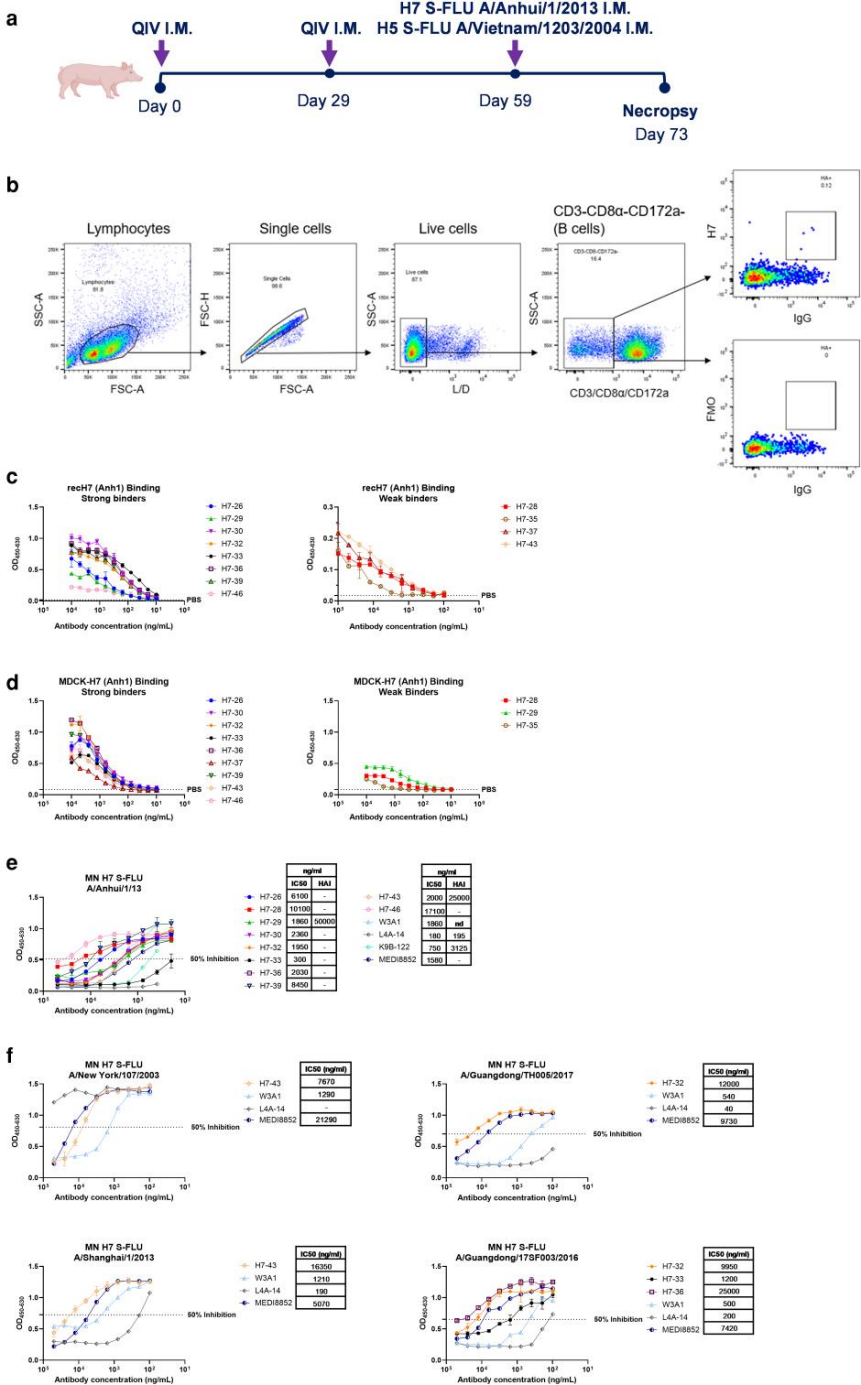
Binding and neutralization of H7-specific porcine mAbs. (a) Experimental design of immunization study. Two 6-weeks old pigs were immunized intramuscularly with 2018/2019 QIV. Twenty-nine days later, the animals were boosted with QIV using the same dose and the route. Thirty days after the boost, animals were immunized with H5N1 S-FLU (H5: A/Vietnam/1203/2004, N1: A/PR/8/34) and H7N1 S-FLU (H7: A/Anhui/1/2013, N1: A/PR/8/34) in equal proportions. Five days post-immunization with S-FLUs, pigs were culled, and tissues were harvested for sorting antigen-specific B cells. (b) Gating strategy to sort single H7-specific IgG positive B cells. H7-specific B cells were sorted in 96-well plate using recH7 protein from A/Anhui/1/2013 (Anh1). (c) Binding to recH7 (Anh1) and binding to (d) MDCK-H7 (Anh1). (e) Concentration of individual mAbs giving 50% neutralization (IC50) against H7 S-FLU A/Anhui/1/2013 and HAI titres. (f) Cross-neutralization titres against distinct avian H7 S-FLU strains. Human mAbs MEDI8852, L4A-14, K9B-122, and W3A1 were used as controls. All assays were performed in at least two independent experiments, each conducted in duplicate. Error bars represent the standard deviation. nd: not determined.

H7-specific mAbs were generated from pigs sequentially immunized intramuscularly with QIV, followed by H5 S-FLU (H5 from VN1203) and H7 S-FLU (H7 from Anh1) as described above for the generation of the H5 mAbs (Fig. 5a). H7-specific B cells were isolated from retropharyngeal lymph nodes using rec H7 protein (Anh1) as a probe (Fig. 5b). A total of 86 single cells were sorted, of which 71 were positive for both IgG heavy and light chains, resulting in 82% recovery. Based on their clustering, twelve antibodies were selected for expression. Among the selected mAbs, five had lambda light chains and seven had kappa light chains ranging from 11 to 21 amino acids length in CDRH3 region (Table 3).

**Table 3.** H7-specific porcine mAbs characterization.

	Gene structure						Binding affinity					neutralization (ng/ml)									Heamaglutination inhibition (ng/ml)	Fc-mediated functions	
H7-specifc mAb	Light chain V family	VH gene family			CDRH3 length (aa)	No. of Cysteines in CDRH3	recH7 Anh1	MDCK-H7	MDCK-H1 Eng195	MDCK-H3 HK5738	MDCK-H5 VN1203	H7 A/Anhui/1/1 3	H7 A/Shanghai/1/13	H7 A/New York/107/2003	H7 A/Netherlands/219/2003	H7 A/Guangdong/TH005/17	H7 Guangdong/17SF003/2016	H7 A/Guangdong/8H324/2017	H7 Taiwan/1/2017	H7 HongKong/125/2017	H7 A/Anhui/1/1 3	CDCC (% Lysis)	NK-degranulation
								Anh1															
H7-26	IGKV2	IGHV1S2*01	IGHD1*02	IGHJ5*01	17	0	+++	+++	+	—	—	6100	—	—	—	—	—	—	—	—	—	++	+
H7-28	IGLV3	IGHV1-14*01, IGHV1S2*01	IGHD1*02	IGHJ5*01	19	0	+	+	—	—	—	10 100	—	—	—	—	—	—	—	—	—	+	+
H7-29	IGLV3	IGHV1-4*02	None assigned	IGHJ5*01	11	0	+++	+	—	—	—	1860	—	—	—	—	—	—	—	—	50 000	+	+
H7-30	IGKV2	IGHV1S2*01	IGHD1*01,IGHD1*02	IGHJ5*01	20	2	+++	+++	—	—	—	2360	—	—	—	—	—	—	—	—	—	++	+
H7-32	IGLV3	IGHV1S2*01	IGHD2*01	IGHJ5*01	17	0	+++	+++	—	—	—	1950	>50 000	—	—	12 000	9950	—	—	—	—	++	+
H7-33	IGLV3	IGHV1S2*01	IGHD1*01,IGHD1*02	IGHJ5*01	16	2	+++	+++	—	—	—	300	—	—	—	—	1200	—	—	—	—	++	+
H7-35	IGKV2	IGHV1S2*01	IGHD1*01,IGHD1*02	IGHJ5*01	19	1	+	+	+	+++	+++	—	—	—	—	—	—	—	—	—	—	+	+
H7-36	IGKV2	IGHV1S2*01	IGHD1*01,IGHD1*02	IGHJ5*01	18	0	+++	+++	++	+++	++	2030	>50 000	—	—	—	25 000	—	—	—	—	++	+
H7-37	IGKV2	IGHV1S2*01	IGHD2*01	IGHJ5*01	21	0	+	+++	+	+++	+++	—	—	—	—	—	—	—	—	—	—	++	+
H7-39	IGKV2	IGHV1S2*01	IGHD1*02	IGHJ5*01	21	2	+++	+++	—	—	—	8450	—	—	—	—	—	—	—	—	—	++	+
H7-43	IGLV8	IGHV1S2*01	None assigned	IGHJ5*01	11	0	+	+++	—	—	—	2000	16 350	7670	>50 000	—	—	—	—	—	25 000	++	+
H7-46	IGKV2	IGHV1S2*01	IGHD1*01,IGHD1*02	IGHJ5*01	17	0	+++	+++	—	—	—	17 100	—	—	—	—	>50 000	—	—	—	—	++	+

For binding affinity recH7 Anh1: —, no detectable binding; +, ≥50 000 ng/ml, weak binding; +++, ≤10 000 ng/ml, strong binding. For binding affinity MDCK-H7 Anh1: —, no detectable binding at starting concentration 10 000 ng/ml; +, OD_450-630_ < 0.5, weak binding; +++, OD_450–630_≥ 0.5, strong binding. For binding affinity to MDCK-H1 (Eng195), MDCK-H3 (HK5738) and MDCK-H5 (VN1203): —, no detectable binding at starting concentration 50 000 ng/ml; +, weak binding, OD_450–630_ < 0.5; ++, intermediate binding, OD_450–630_ ≥ 0.5 & < 1.0; +++, strong binding, OD_450–630_ ≥ 1.0. For neutralizing activity, —, non-neutralizing; and for neutralizers, the concentration at which 50% of the viruses are inhibited, defined as the IC_50_, is indicated. Haemagglutination-inhibition activity is defined as the minimal concentration of the antibody required to prevent haemagglutination. For CDCC: —, no detectable lysis; +, detectable lysis at starting mAb concentration of 40 ug/ml; ++, detectable lysis at starting mAb concentration of 10 ug/ml. For ADCC: +, <30% CD107a expressing NK cells; ++, ≥ 30% CD107a expressing NK cells. nd: not determined.

H7-specific mAbs were assessed for binding to rec H7 (Anh1) and MDCK cells expressing H7 (Anh1). Among the 12 H7-specific mAbs tested, 8 (H7-26, H7-29, H7-30, H7-32, H7-33, H7-36, H7-39, and H7-46) exhibited strong binding to rec H7 and 4 (H7-28, H7-35, H7-37, and H7-43) showed weak binding (Fig. 5c, Table 3). Binding to MDCK-H7 cells was observed for nine mAbs (H7-26, H7-30, H7-32, H7-33, H7-36, H7-37, H7-39, H7-43, and H7-46) while three (H7-28, H7-29, and H7-35) showed weaker (OD value <0.5) binding at the highest concentration of 10 000 ng/ml (Fig. 5d). MAbs H7-37 and H7-43 demonstrated stronger binding to MDCK-H7 (Anh1) than to rec H7 (Table 3).

The neutralizing activity of strong and weak binders was first evaluated at a concentration of 50 µg/ml. Neutralizers were further analysed to determine their IC50 values. Ten H7 mAbs (H7-26, H7-28, H7-29, H7-30, H7-32, H7-33, H7-36, H7-39, H7-43, and H7-46) showed neutralizing activity against the homologous Anh1 ranging from 300 to 17 100 ng/ml, with H7-33 exhibiting strong neutralizing activity at IC50 300 ng/ml (Fig. 5e). Neutralizing mAbs were further evaluated for their HAI activity. Two mAbs, H7-29 and H7-43, demonstrated HAI at 50 and 25 μg/ml respectively (Figure 5e). Interestingly, although H7-32 exhibited broad neutralizing activity, it did not inhibit haemagglutination, suggesting that it may target a conserved stem epitope. Cross-neutralization was analysed against eight avian H7 viruses (A/Shanghai/1/13, A/NewYork/107/2003, A/Netherlands/219/2003, A/Guangdong/TH005/17, A/Guangdong/17SF003/2016, A/Guangdong/8H324/2017, A/Taiwan/1/2017, and A/Hong Kong/125/2017). MAbs H7-32 and H7-43 neutralized three distinct H7 strains. MAb H7-32 neutralized three H7 strains (H7 A/Shanghai/1/13, H7 A/Guangdong/TH005/17, and H7 Guangdong/17SF003/2016) and H7-43 neutralized H7 A/Shanghai/1/13, H7 A/New York/107/2003, and H7 A/Netherlands/219/2003 (Fig. 5f, Table 3).

**Figure 6:**
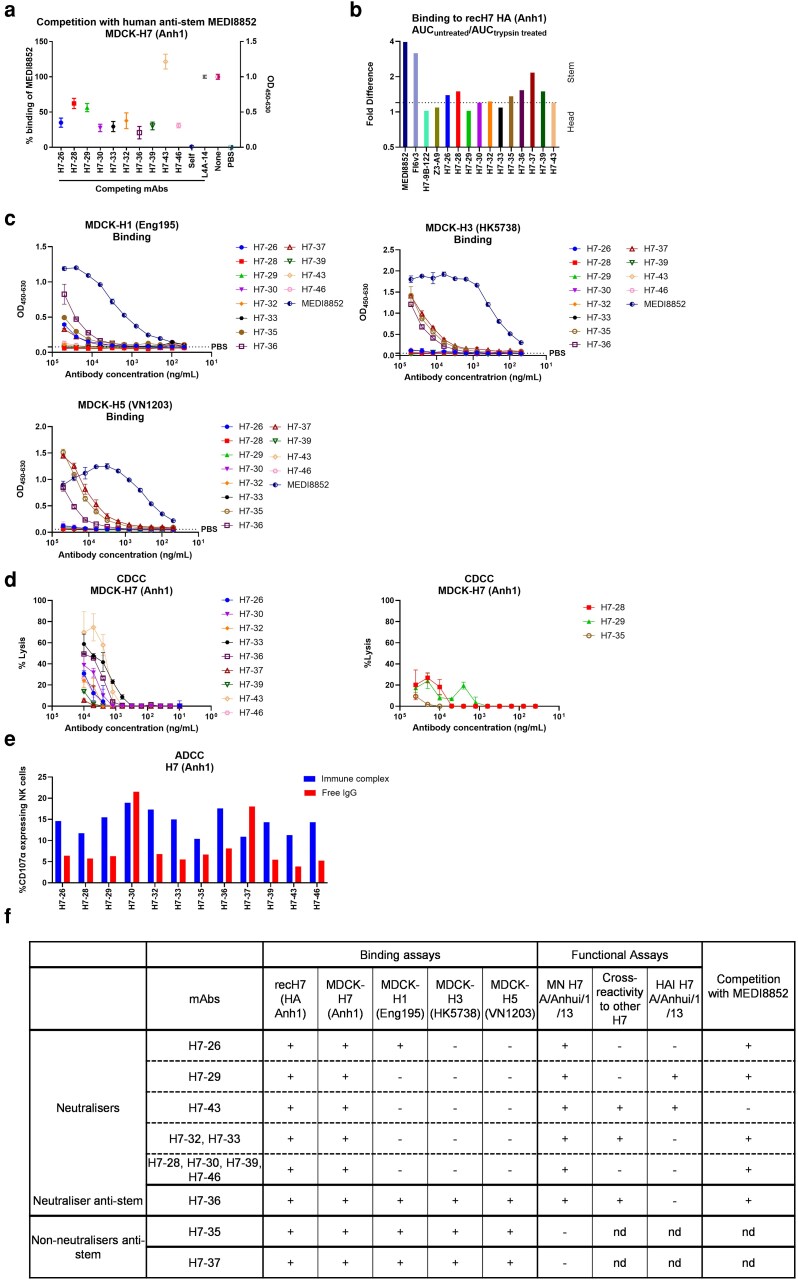
Cross reactivity and Fc-mediated functions of H7-specific porcine mAbs. (a) Competition of H7 mAbs with MEDI8852 for binding to MDCK-H7 (Anh1) cells. (b) Fold difference of binding of mAbs to recombinant HA and trypsin-treated HA. Area under curve of titration points were measured to derive the fold difference. MEDI8852 and FI6v3 are known stem mAbs, whereas H7-9B-122 and Z3-A9 are known head mAbs. The line at 1.2 is an arbitrary cut-off. (c) Cross-reactivity with MDCK-H1 Eng195 (HA A/England/195/2009), MDCK-H3 (HK5738), and MDCK-H5 (VN1203) cells. (d) Complement-dependent cell cytotoxicity (CDCC) with MDCK-H7 (Anh1) cells incubated with H7 mAbs in presence of rabbit complement. CDCC activity was measured by LDH release. (e) ADCC was quantified by percentage of NK cells expressing CD107a. (f) Summary table for H7 mAbs. nd: not determined. Error bars represent the standard deviation.

Other neutralizing but HAI-negative mAbs included H7-26, H7-28, H7-30, H7-32, H7-33, H7-36, H7-39, and H7-46, suggesting that they might be potential broadly cross-reactive anti-stem antibodies. We therefore analysed the inhibition of binding of anti-stem MEDI8852 to MDCK-H7 (Anh1) by these eight H7 mAbs. MAbs H7-26, H7-30, H7-32, H7-33, H7-36, H7-39, and H7-46 inhibited the binding of MEDI8852 by 62–80% (Fig. 6a). An additional assay was performed by comparing mAbs binding to recombinant soluble H7 HA and its trypsin-treated form, in which HA0 trypsin-cleavage specifically reduce HA2/stem mAb binding, to distinguish head- from stem-directed mAbs. MAbs H7-26, H7-28, H7-35, H7-36, H7-37, and H7-39 showed reduced binding to trypsin-treated HA suggesting these to be stem mAbs (Fig. 6b). To further investigate the anti-stem properties, we tested the binding of these potential anti-stem mAbs to MDCKs expressing either H1 (Eng195), H3 (HK5738), or H5 (VN1203) and showed that H7-36 bound to all (Fig. 6c). Interestingly the non-neutralizing mAbs H7-35 and H7-37 bound to MDCK-H1, MDCK-H3, and MDCK-H5 (Fig. 6c). This cross-reactivity, together with MEDI8852 competition and lack of HAI, suggests that they are directed to stem epitopes.

MAbs that showed strong binding to MDCK-H7 (Anh1) cells were evaluated for their Fc-mediated functions. Among these, H7-33 and H7-43 demonstrated robust CDCC responses, inducing up to 80% cell lysis at a concentration of 10 µg/ml (Fig. 6d). H7 mAbs were not as potent in inducing NK cell activity compared to H3 and H5-specific mAbs, inducing less than 20% NK cell activity. Interestingly, H7-30 and H7-33 exhibited equal or greater NK cell degranulation as free IgG compared to their immune complex form (Fig. 6e).

Overall, these results show that a third of the H7-specific mAbs inhibited the binding of MEDI8852 to MDCK-H7 (Anh1) cells indicating that the mAbs might be recognizing stem epitopes (Fig. 6f). Four of them (H7-32, H7-33, H7-36, and H7-43) exhibited broadly neutralizing activity. MAb H7-36 bound to MDCK-H1 (Eng195), MDCK-H3 (HK5738), and MDCK-H5 (VN1203) cells suggesting that it recognizes both group 1 and group 2 influenza viruses. Another group of H7 mAbs strongly bound to the rec H7 (Anh1) and MDCK-H7 (Anh1) cells and neutralized the immunizing Anh1 strain. Interestingly only two mAbs (H7-29 and H7-43) inhibited haemagglutination at a very high concentration. The third group of mAbs (H7-35 and H7-37) did not neutralize but bound to MDCK-H1 (Eng195), MDCK-H3 (HK5738), MDCK-H5 (VN1203), and MDCK-H7 (Anh1) cells, suggesting that they are targeting stem epitopes.

## Discussion

Monoclonal antibodies are powerful tools for understanding viral evolution, guiding therapeutic development, and informing rational vaccine design. The generation of both strain-specific and broadly reactive mAbs is also an important step for improving preparedness against influenza viruses with pandemic potential. Here, we describe the first to our knowledge porcine monoclonal antibodies, directed against H3, H5, and H7 influenza haemagglutinins. These mAbs provide new tools for understanding influenza immunity in pigs, a key intermediate host.

Vaccine effectiveness against H3N2 in 2016–18 was reduced due to egg-adaptive mutations, particularly T160K and L194P within antigenic site B, the immunodominant epitope of the HA head [22, 23, 28]. In the present study, pigs were inoculated intranasally with an egg-derived H3N2 A/HK/4801/2014 virus containing these adaptive mutations. Similarly to vaccinated humans, pigs generated an antibody, H3-57, that bound L194P and specifically recognized the egg-adapted A/HK/4801/2014 virus but not rec H3 (HK5738) that matches the circulating strain. This antibody was identified incidentally, despite sorting with rec H3 (HK5738) bait protein with leucine at position 194 and not carrying any egg-adaptive mutation. Had the egg-adapted rec HA carrying both T160K and L194P mutation been used as the bait, it is likely that more site B-specific antibodies would have been isolated.

Among the isolated H3-specific mAbs, dominance to site B or to other classical sites A–E was not observed [29, 30]. Instead, these pig mAbs appear to target other HA head key residues conserved between egg-derived and wild-type virus. A limitation of this study is that neutralization was not assessed against additional substitutions within classical head or stem epitopes, and mapping is incomplete, precluding detailed comparison with the human/mouse H3 antibody repertoire. However, while humans often show immunodominance to site B due to priming and exposure history, responses to alternative head sites are also observed [31].

The widespread distribution and expanding host range of high pathogenic avian influenza viruses (HPAIVs) make them a major threat to wildlife, livestock, and humans, with significant implications for food security and zoonotic risk. Although H5 and H7 viruses are generally not considered pig pathogens, H5 seropositivity has been reported, and experimental infection with mammalian adapted H5N1 by intratracheal inoculation has been demonstrated [32]. If HPAIV were to spread beyond cattle and into pigs, the consequences for agriculture and public health could be severe. Since pigs are not naturally infected with H5 or H7 viruses, we used sequential immunization with QIV, H5-S-FLU, and H7-S-FLU, demonstrating that both H5- and H7-specific antibodies with neutralizing activity can be induced. A significant proportion of H5- and H7-specific mAbs showed both binding and neutralizing activity, while some exhibited binding without neutralization. Head antibodies tend to be better neutralizers by targeting the receptor binding site but can also induce the Fc-mediated mechanism. In contrast, stem mAbs tend to be weak or moderate neutralizers and heavily depend on the Fc-mediated functions *in vivo* for their protective effects. In this study, Fc-mediated functions correlated with the strength of antibody binding rather than with stem or head epitope specificity.

Several H5 mAbs inhibited haemagglutination, indicating recognition of HA head epitopes. However, as relatively few H5 mAbs have been mapped, our ability to precisely define their epitopes was limited. Among the H7 mAbs, several (H7-32, H7-33, H7-36, and H7-43) mAbs demonstrated broad neutralizing activity against diverse H7 viruses between 2013-2019 spanning two lineages—Yangtze River Delta, the Pearl River Delta and both low pathogenic and high pathogenic avian viruses. Some of these mAbs also inhibited binding of the broadly neutralizing human mAb MEDI8852, suggesting recognition of the HA stem. Binding studies with recombinant soluble H7 HA and its trypsin-treated form further confirmed that H7-35, H7-36, and H7-37 are anti-stem antibodies. Importantly, H7-35, H7-36, and H7-37 bound MDCK cells expressing H1, H3, and H5 HAs, demonstrating cross-group reactivity and that porcine anti-stem mAbs targeting both group 1 and group 2 HAs can be generated.

Although the stem is less immunogenic than the globular head, anti-stem antibodies can confer broad protection due to their conserved nature making it an attractive vaccine target. Sequential immunization with chimeric HAs has been used to redirect responses towards the stem [33], but in pigs such vaccines did not induce anti-stem responses and only provided partial protection against H1N1. A possible explanation for the different results from our study is that the pigs used had maternal antibodies, which may have prevented the induction of potent antibody responses [33]. Recently, porcine mAbs against the influenza HA stem were generated from pigs immunized with recombinant H1N1 HA protein and their epitopes identified [34]. One of the group cross-reactive mAbs targeted the well-characterized central stem epitope akin to MEDI8852, while another bound to a linear epitope spanning the HA1/HA2 junction, revealing pig-specific binding motifs [34]. Further studies will be required to determine the precise epitopes recognized by the mAbs described in the present paper.

In humans, stem epitopes are subdominant and usually only emerge after repeated heterologous exposures. In our pig study, sequential heterologous immunization (QIV followed by H5- and H7-pseudotyped viruses) may have favoured the recall of rare, conserved stem-specific B cells seeded by the initial HA exposures, e.g., the group 1 and 2 cross-reactive mAb H7-36. However, because pigs were naïve to H5 and H7, the head-directed and non-cross-reactive stem antibodies likely arose from *de novo* B-cell responses. Interestingly, more stem-reactive mAbs were recovered against H7 than H5, even though the H5 and H7 were administered simultaneously following QIV priming. The reasons for this imbalance are not certain but may include preferential presentation of the H7 stem epitope compared to H5, thereby favouring selection of B cells producing antibodies to the H7 stem, or stochastic variation in precursor frequency and B-cell recruitment, or potential bias introduced during the B-cell sorting procedure.

The antigen-specific B cells from which the mAbs were generated were isolated from draining lymph nodes rather than blood as routinely done in humans. Tracheobronchial lymph nodes draining the lungs were sampled following H3N2 or H1N1 infection, while retropharyngeal lymph nodes draining the neck muscles were used after intramuscular immunization with QIV, H5-S-FLU, or H7-S-FLU. In human and mouse, germinal-centre-associated lineages are more dynamically evolving (thus more clonal and epitope diversification) compared to peripheral B cells [35, 36]. Whether the route of antigen exposure or sampling location shaped the antibody repertoire remains to be determined.

Porcine mAbs have been developed against several major swine pathogens, including porcine epidemic diarrhoea virus, porcine delta coronavirus, foot-and-mouth disease virus, classical swine fever virus, porcine reproductive and respiratory syndrome virus, African swine fever virus, and hepatitis E virus [37–42]. These mAbs are valuable tools for investigating passive immunotherapy, epitope mapping and diagnostic development, and facilitate vaccine design by identifying conserved neutralizing sites capable of inducing cross-protective immunity. Although currently at the experimental stage, they represent important advances in swine disease control with potential translational relevance for zoonotic infections.

In contrast, influenza mAb development has advanced considerably, with several broadly neutralizing antibodies entering clinical trials and guiding vaccine design. Stem mAbs such as VIS410, MHAA4549A, VIR-2482, and CR9114 [43–45] target conserved epitopes on the HA, neutralize diverse influenza A strains, and are being evaluated for both therapeutic and prophylactic use. Pigs, as large natural hosts of influenza with close physiological similarities to humans, offer a valuable model for antibody discovery. In this study, we generated the first porcine mAbs against H3, H5, and H7 influenza viruses following infection or immunization and demonstrated that pigs recognize key egg-adapted H3N2 mutations, as do humans, with some directed against the HA stem. These porcine mAbs provide important new tools to investigate influenza virus evolution, explore therapeutic applications, and assess pharmacokinetics in a model more comparable to humans than small animals.

## Materials and methods

### Viruses, vaccines, MDCK cell lines, and rec proteins

#### MDCK-SIAT1 cells expressing HA and S-FLU viruses

cDNA encoding haemagglutinin was human codon-optimized and synthesized by GeneArt or Genscript. Lentiviral vector pHR-SIN was engineered to incorporate the HA cDNA. Lentivirus was produced by co-transfection of HEK 293T cells with a lentiviral vector and VSV-G and gag-pol expressing plasmids. MDCK-SIAT1 cells were transduced with lentivirus in presence of polybrene (1 µg/ml), to express full-length HA. The HA transduced cells were sorted using flow cytometry following detection by HA-specific mAb.

The development of S-FLU, nonreplicating pseudotyped influenza virus, has been described previously [46]. The seed S-FLU viruses were used to infect a monolayer of MDCK-SIAT1 cells stably expressing the full-length HA. The virus was grown in presence of 1 µg/ml TPCK-Trypsin (Sigma) and harvested 2 days post-infection. The viruses were stored at −80 °C. They were titrated on MDCK-SIAT1 cells for use in microneutralization assays. The following stable cell lines and the S-FLU pseudotyped viruses were prepared [46]; H1 A/England/195/09, H3 A/Hong Kong/5738/14, H5 A/Vietnam/1203/2004, H5 A/Texas/37/2024, H7 A/Anhui/1/13, H7 A/Shanghai/1/13, H7 A/New York/107/2003, H7 A/Netherlands/219/2003, H7 A/Guangdong/TH005/17, H7 A/Guangdong/17SF003/2016, H7 A/Guangdong/8H324/2017, H7 A/Taiwan/1/2017, and H7 Hong Kong/125/2017.

#### Recombinant HAs

The HA gene (residues 1–521) was fused at its C-terminus to a foldon trimerization domain, a flexible linker, a SpyTag, and a hexahistidine purification tag. The polybasic cleavage site in H5 HA (VN1203) was altered: QRERRRKKR↓G to QRETR↓G (where the arrows indicate cleavage sites) [47]. This fusion construct was cloned into the pCDNA3.1(−) vector between NotI and EcoRI restriction sites. Transient transfection of Expi293 cells (Thermo Fisher A14635) was performed according to the manufacturer’s protocol. Five to 7 days post-transfection, the culture supernatant was harvested and clarified by centrifugation. The clarified supernatant was loaded onto a HisTrap HP column (Cytiva 17524701) equilibrated in phosphate-buffered saline (PBS; 20 mM sodium phosphate monobasic dihydrate, 0.5 M NaCl 20 mM Imidazole, pH 7.4). Bound protein was eluted with tris-buffered saline containing 0.5 M imidazole then desalted to PBS buffer using ZebaSpin desalting columns (Thermo Fisher 89893). Aliquots of the HA protein were stored at −80°C until use.

#### Viruses

H3N2 A/Hong Kong/4801/2014 (virus obtained from the NIBSC, UK) and H1N1pdm09 (A/Michigan/45/2015) (pH1N1) were propagated in MDCK cells.

#### Vaccines

QIV (A/Michigan/45/2015 (H1N1) pdm09-like virus, an A/Singapore/INFIMH-16-0019/2016 (H3N2)-like virus, a B/Colorado/06/2017-like strain, a B/Phuket/3073/2013-like strain, and 15 microgram haemagglutinin per strain per 0.5 ml dose).

#### Monoclonal antibodies

Anti-H5 HA head mAb 21D85A is a generous gift from Professor Kuan-Ying Huang (National Taiwan University). MEDI8852 mAb is manufactured in-house using the variable sequences obtained from PDB 5JW4.

### Animal studies

All experiments were approved by the ethical review processes at the Pirbright Institute and conducted according to the UK Government Animal (Scientific Procedures) Act 1986 under PPL P47CE0FF2. The Pirbright Institute conforms to ARRIVE guidelines. For all studies, pigs were obtained from a commercial high-health status herd, based on routine health surveillance, regular pathogen monitoring, scheduled health checks, comprehensive health records, documented absence of clinical disease, and the implementation of strict biosecurity measures, including vaccination programs and hygiene protocols. The pigs were screened by ELISA for the absence of serum antibodies against A/swine/England/1353/2009 (pH1N1). In all experiments, animals acclimatized for at least 7 days and were randomized into different groups and pens using Excel by the animal services staff. Researchers processing the samples were only aware of the pig numbers, not the group assignment.

#### Generation of H3 mAbs

Six female Landrace × Hampshire cross pigs, each 5 weeks old, were obtained from a commercial high-health status herd. Pigs were intranasally infected with 2 ml per nostril of H3N2 (A/Hong Kong/4801/2014) at a concentration of 1 × 10^7^  *PFU*/ml using a mucosal atomisation device (MAD300) [48]. Three weeks later, all pigs were challenged with 2 ml per nostril of H1N1 (A/Michigan/45/2015) at the same concentration. Four days post challenge with H1N1, all pigs were humanely culled with overdose of pentobarbital sodium anaesthetic (Fig. 1a) [48]. Tracheobronchial lymph nodes were collected and processed as previously described [49] to isolate H3-specific B cell. We have previously shown that TBLN are reliable source of antigen-specific B cells [20].

#### Generation of H5 and H7 mAbs

Two 6–8 weeks old female Landrace pigs were obtained from a commercial high-health status herd. All the pigs were immunized intramuscularly with two human doses (2 ml per pig) of 2018/2019 season quadrivalent influenza vaccine (QIV) (inactivated, split) (Sanofi, Lot no: R4A331 V). Twenty-nine days later, the animals were boosted with QIV using the same dose and route. Thirty days after the boost, animals were immunized intramuscularly with 2 × 10^8^ TCID_50_/ml of H5N1 S-FLU (H5: A/Vietnam/1203/2004, N1: A/PR/8/34) and 2 × 10^8^ TCID_50_/ml of H7N1 S-FLU (H7: A/Anhui/1/2013, N1: A/PR/8/34) in equal proportions [50]. Five days post immunization with S-FLUs, pigs were humanely culled, and retropharyngeal lymph node were harvested for sorting of antigen-specific B cells.

### Single-cell sorting for H3, H5, and H7-specific B cells

Cryopreserved cell suspensions from the TBLN (H3) or RPLN (H5 and H7) were thawed, counted, and filtered using 70 µm cell strainer. Cells were stained with CD3-RPE (Clone: BB23-8E6-8C8, BD Bioscience), CD8α-RPE (Clone: 76-2-11, BD Bioscience), CD172α-RPE (Clone:74-22-15, BIO-RAD, UK), near-IR fixable Live/Dead stain (Invitrogen, UK), and either biotinylated H3 (HK/5738/14), biotinylated H5 (A/VN1203/04) or biotinylated H7 (A/Anhui/1/2013) for 30 min at 4°C. Cells were washed with PBS twice before staining with: Streptavidin-BV421 (Cat: 563259, BD Bioscience), IgG (H + L) AF 647 (Mouse anti-pig IgG (H + L), Abbexa) for 30 min at 4°C. The cells were washed three times, re-suspended with pre-chilled 0.5% BSA+PBS and passed through a 70 μm cell strainer (BD Biosciences, UK) prior to cell sorting. Single colour controls were used for compensation and fluorescence minus one primary Ab controls were used to set thresholds.

HA-specific B cells were sorted, using DIVA 8 acquisition software and a FACS Aria III cell sorter (BD Biosciences), at single-cell density into hard shell 96-well PCR plates (BIO-RAD, UK) containing 10 μl/well of RNA catch buffer (10 mM Tris pH8.0 supplemented with RNasin 10 000 units (Promega, UK). The FACS gating strategy to identify HA positive, single B cells are shown in Figs. 1b, 3b and 5b. In brief, samples were gated on lymphocytes (SSC-A vs FSC-A), singlets (FSC-H vs FSC-A), followed by the identification of live cells by a live/dead stain. HA-specific IgG cells were then determined as CD3^−^, CD8α^−^, CD172α^−^ and double positive for the receptive HA and IgG. Non-specific binding was controlled by gating on mesenteric lymph node cells. After sorting, plates were immediately covered with aluminium foil seals, centrifuged for 5 min at 300 × g, 4˚C, and transferred to −80˚C.

### Amplification and indexing of heavy and light chains from single cells

cDNA synthesis on single sorted cells was carried out in total reaction volumes of 20 µl using Sensiscript reverse transcriptase (205213 Qiagen, UK) containing a mix of oligonucleotides dT_23_VN (NEB, UK), random primers (NEB, UK), and Rnaseout 10 U (Thermo Fisher). The cDNA reaction was incubated for 60 min at 37˚C. cDNA was synthesized in one reaction, and aliquots of the cDNA were used in subsequent PCRs to amplify the variable heavy and light chains regions in separate reactions. A nested PCR approach was chosen to have enough materials for the downstream indexing for next-generation sequencing. Reverse single-cell transcription of RNA and nested PCR of porcine IgG, Igκ, and Igλ genes were adapted from a previously described protocol [51], with new primers being designed for porcine variable and constant regions of the IgG heavy and light chain loci. Four microliters of cDNA were used to amplify heavy or light chain in two steps of PCRs as shown in Supplementary Table S1. PCR I products were used as the template for a second round of PCR (PCR II) reactions in which overhang (-OH) Illumina adapter sequences were added to primers as anchors for the indexing [51]. The PCR amplification was performed as shown in Supplementary Table S2. The PCR II products were purified with AMPure XP beads (Beckman Coulter Life Sciences; Indianapolis, IN, USA) using a 0.65 bead/amplicon volume ratio following the manufacturer’s protocol. PCR II products were indexed using 10 cycles amplification PCR as shown in Supplementary Table S2. The final products were purified using the AMPure XP beads.

### Sequencing and bioinformatic analysis

Amplicons from individual samples were analysed using a TapeStation 4200 system with DNA D1000 ScreenTape (Agilent Technologies, Inc., Santa Clara, CA, USA). Samples showing a distinct band around 500 bp were pooled to a final concentration of 5 nM. The pooled library was quantified using the NEBNext Library Quantitation Kit for Illumina (New England Biolabs, Ipswich, MA, USA), with concentration confirmation performed on a Qubit fluorometer (Thermo Fisher Scientific, Waltham, MA, USA). Sequencing was carried out on Illumina platforms (Illumina Inc., San Diego, CA, USA), following the manufacturer’s protocol and incorporating a 40% PhiX control to increase sequence diversity. Libraries prepared from H5 and H7 single cells were sequenced on an Illumina MiSeq instrument, while libraries from H3 single cells were sequenced on the Illumina NextSeq. Bioinformatics analysis was performed as previously described [51]. Initial quality control of raw data was performed using the FastQC v0.12.1 tool. Subsequent filtering (Q≥30) and adapter trimming were carried out using Trim Galore! v0.6.10 to ensure high-quality sequence input. Filtered paired-end reads were merged into contiguous sequences using the FLASH v1.2.11 tool and assembled sequences were then converted to fasta format using **seqtk v1.4-r122} for downstream bioinformatic analysis, as previously described. Briefly, the sequences were analysed for comprehensive V(D)J assignment, isotype determination, and framework annotation utilizing Sus scrofa IMGT reference (https://www.imgt.org/download/V-QUEST/IMGT_V-QUEST_reference_directory/; 2024) using IgBLASTn (v1.22.0, 2024). Framework annotation was conducted using IgMAT (Immunoglobulin sequence Multi-species Annotation). Single-cell sequencing analysis was performed using USEARCH v11.0.667. For each single cell, antibody sequences were retrieved from the most abundant heavy and light chain sequences (threshold >70%). The complementarity-determining region 3 of the heavy chain (CDRH3) was extracted from the selected high-confidence pairs. CDRH3 sequences were then clustered using UCLUST with a 94%-identity threshold. Final antibody candidates were selected from these distinct CDRH3 clusters, with selection criteria prioritizing the unique CDRH3 sequence and its corresponding V gene usage to ensure diversity of selected sequences.

### Cloning, expression and purification of mAbs

The recombinant antibodies were produced as described previously [52]. Briefly the light and heavy chain variable regions for the selected H3-, H5-, and H7-specific monoclonals were ordered as synthetic gene fragments from Twist Bioscience (Twist Bioscience, California, USA) and directionally cloned by in-fusion (Vazyme, Nanjing, China, C112-01) into the pNeoSec_SS_VL_kappa, pNeoSec_SS_VL_lambda, and pNeoSec_SSFc_IgG1 vectors (Immunological Toolbox, The Pirbright Institute, UK). The antibodies were transiently expressed in Expi293FTM cells according to Gibco Expi293™ Expression System User Guide (Thermo Fisher Publication Number: MAN0007814). Briefly 50 µg of heavy chain and 50 µg of the corresponding light chain plasmids were mixed in Expi293 medium containing 0.5% of polyethylenimine (PEI MAX; Polysciences, Generon, UK) and incubated at room temperature for 10 min before transfecting 100 ml of 2 × 10^6^ vc/ml Expi293FTM cells cultured overnight at 37 °C with 5% CO_2_ on an orbital shaker at 120 rpm in 250 mL Erlenmeyer flasks. Sixteen hours post-transfection, valproic acid (0.76 mg/ml), sodium propionate (0.61 mg/ml), and glucose (0.75%) were added to the culture. Four days after transfection, cell supernatants were harvested by centrifugation at 2000 × g for 10 min and filtered through a 0.22 μm membrane (Merck, UK) for downstream antibody purification.

Antibodies were purified from culture supernatants using a 5 mL HiTrap™ Protein G HP column mounted on an ÄKTA start™ chromatography system (Cytiva, UK). The supernatant was passed through the column, and bound antibodies were eluted with 0.1 M glycine-HCl, pH 2.7. Eluted fractions were immediately neutralized with 1/10th volume of 1 M Tris-HCl, pH 8.0. The purified antibodies were then dialysed overnight against PBS using 10 kDa molecular weight cut-off dialysis cassettes (Thermo Fisher Scientific, UK). Antibody purity and heavy/light chain expression were evaluated by native and denaturing electrophoresis using NuPAGE™ 4–12% Bis-Tris gels (Thermo Fisher Scientific, UK).

### Binding assays

#### Recombinant HA protein ELISA

Maxisorp 96-well plates (Thermo Scientific, UK) were coated with 1 µg/ml of either of the recombinant HA proteins: rec H3 A/HK/5738/14, rec H5 A/VN/1203/2004, or rec H7 A/Anhui/1/2013 in PBS and incubated overnight at 4 °C. Plates were washed with PBS containing 0.05% Tween-20 (PBS-T) and blocked with 4% (w/v) semi-skimmed milk in PBS-T for 2 h at room temperature (RT). Antibodies were serially diluted in blocking buffer, and 100 µl per well was added and incubated for 1 h at RT. After three washes with PBS-T, goat anti-pig IgG (H + L) HRP-conjugated secondary antibody (Bio-Rad, 1:20 000 dilution in blocking buffer) was added for 1 h at RT. Plates were washed three times with PBS-T and developed with 50 µl per well of 3,3′,5,5′-tetramethylbenzidine (TMB) substrate (BioLegend). The reaction was stopped with 50 µl of 1 M H_2_SO_4_. Optical density (OD) was measured at 450 nm and 630 nm using an Absorbance Microplate Reader (BioTek, Swindon, UK).

#### MDCK-HA

MDCK-SIAT1 cells expressing HA subtypes H1, H3, H5, or H7 were seeded at 3 × 10^6^ viable cells per plate in Nunc MicroWell 96-well plates (Thermo Scientific, UK) using complete DMEM supplemented with 10% foetal calf serum and 100 IU/ml penicillin. Cells were incubated overnight at 37°C with 5% CO_2_. Prior to antibody incubation, wells were washed with PBS. Fifty microliters of serially diluted monoclonal antibodies in dilution buffer (PBS containing 0.1% BSA) were added and incubated for 1 h at RT. Plates were washed twice with PBS, followed by a 1 h incubation with goat anti-pig IgG (H + L) HRP-conjugated secondary antibody (Bio-Rad, 1:20 000 dilution in dilution buffer). Plates were then washed and developed using TMB substrate as described above.

### HA ELISA to differentiate head and stem mAbs

Recombinant H7 HA (4 µg/ml) was coated onto Nunc 96-well microplates. HA was treated with 2.5 µg/ml TPCK-trypsin (*N*-tosyl-L-phenylalanine chloromethyl ketone; Sigma-Aldrich, T1426) in PBS for 15 min at room temperature to cleave HA0 into HA1 and HA2 polypeptides. During this process, free soluble HA may undergo conformational changes in HA2, potentially affecting the binding of stem-directed monoclonal antibodies (mAbs). Following trypsin treatment, plates were blocked with 5% milk. mAbs were titrated starting at 50 µg/ml using an 8-point, 5-fold serial dilution and incubated with both treated and untreated HA. Known head- and stem-specific human mAbs were included as controls. Binding of human mAbs was detected using HRP (horseradish peroxidase)-conjugated rabbit anti-human IgG (Dako, P0214; 1:2000), while pig mAbs were detected using HRP-conjugated anti-pig IgG (Invitrogen, PA1-28602; 1:10 000). Plates were developed with TMB (3,3′,5,5′-tetramethylbenzidine) substrate (SeraCare, 5120-0077), and reactions were stopped with 1 M H_2_SO_4_. Optical density was measured at 450 nm with a 630 nm reference using a Clariostar plate reader (BMG Biotech). The area under the titration curve (AUC) was calculated from log-transformed mAb concentrations in GraphPad Prism. For analysis, the ratio of AUC values for H7-specific mAb binding to untreated versus trypsin-treated HA was determined, with higher ratios indicating stem-directed mAbs.

### Virus neutralization and haemagglutination inhibition assays

Neutralizing antibody titres were determined by microneutralization assay (MN) on MDCK sialyltransferase (SIAT1) cells as described previously [52]. Antibodies were serially diluted in viral growth media (DMEM with 0.1% BSA, 10 mM HEPES, 100 IU/ml penicillin, 2 nM Glutamine). Viruses were titrated beforehand in the absence of antibodies to determine the *PFU*/ml necessary to yield a plateau infection overnight in MDCK-SIAT1 cells for the MN assay [21]. Viruses were then added to the diluted antibody samples in equal volume and incubated at 37°C with 5% CO_2_ for 2 h. The MDCK-SIAT1 cells were prepared as single-cell suspension and added to the sera and virus mixture, and the plates were further incubated for 18 h. The cell layer was fixed with 4% paraformaldehyde, washed with PBS and permeabilized with 0.05% Triton-X100 and 20 mM glycine. The cells were stained with anti-NP (clone: AA5H; Bio-Rad Laboratories) followed by goat anti-mouse HRP (Dako). Finally, plates were developed with 50 μl TMB substrate solution (Biolegend) and reaction was stopped with 50 μl of 1 M H_2_S0_4_. Optical density at 450 and 630 nm was measured with the Absorbance Microplate Reader (Biotek, Swindon, UK). IC50 (concentration of added antibody in 50 µl that suppressed S-FLU expression by 50% as measured by NP expression) was calculated by linear interpolation.

HAI was carried out using chicken red blood cells (1% vol/vol) and S-FLU viruses at a concentration of 4 HA units/25 μl, as described in World Health Organization Manual on Animal Influenza Diagnosis and Surveillance (WHO/CDS/CSR/NCS/2002.5 Rev.1).

For both HAI and MN assay, previously characterized human monoclonal antibodies against H7-head (4A-14, K9B-122, and W3A1), H5-head (21D85A) and pan-stem (MEDI8852), were used as controls [46].

### Competition binding assay

For epitope mapping, neutralizing H3, H5, and H7-specific antibodies, as determined by MN and HAI, were tested for inhibition of binding of known human pan-stem MEDI8852. Briefly, MDCK-H3 (HK5738) and (HK5738 L194P) for H3 mAbs, MDCK-H5 (VN1203) for H5 mAbs and MDCK-H7 (Anhui1) for H7 mAbs were seeded in Nunc MicroWell 96-Well Microplates (Thermo Scientific, UK) at a density of 3 × 10^6^ viable cells per plate in complete DMEM (supplemented with 10% FCS and 100 IU/ml penicillin) and incubated overnight at 37°C with 5% CO2. The following day, the cells were washed with PBS before applying of mixture made of 1 μg/ml of the biotinylated human mAb and 20 μg/ml of porcine mAb to the cells and incubating for 1 h at RT. Cells were subsequently washed twice with PBS and binding of the MEDI8852-biotin was detected using Ultra Streptavidin (Invitrogen) diluted at 1:1000 in dilution media. After 1 h incubation, plates were washed twice with PBS and developed with 50 μl TMB substrate solution (Biolegend) and reaction was stopped with 50 μl of 1 M H_2_S0_4_. Optical density at 450 and 630 nm was measured with the Absorbance Microplate Reader (Biotek, Swindon, UK). The binding (%) of MEDI8852-biotin was calculated as (X- Min)/(Max-Min)*100 where X = measurement of the competing porcine mAb, Min = binding of MEDI8852-biotin in the presence of non-conjugated MEDI8852, Max = binding of MEDI8852-biotin in presence of a head human mAb. Means and 95% confidence intervals for eight replicates are shown.

### Complement-dependent cellular cytotoxicity

CDCC activity of mAbs was measured using rabbit low-tox-H complement (Cedarlane Laboratories) as described previously [52]. Briefly, rabbit sera were pre-adsorbed on MDCK cells expressing respective HA against the mAbs (HK5738 and HK5738 L194P for H3 mAbs, VN1203 for H5 mAbs and Anhui1 for H7 mAbs) for 30 min at 4°C. Adsorbed sera were harvested by spinning down the cells pellet. MDCK-HA cells were suspended in AIM-V medium at concentration of 3 × 10^5^ cells/ml. One hundred microliters of cells were incubated with 50 µl per well of serially diluted mAbs for 10 min at RT and mixed with pre-adsorbed complement at a final concentration of 5% for 2 h at 37°C. Cells were spun at 420×g for 4 min and 50 µl of supernatant was transferred to a flat bottom plate to which 50 µl of LDH-substrate (Roche Diagnostics) was added to measure the released lactate dehydrogenase (LDH) for lysed cells due to complement activation. The plate was read with the kinetic protocol (8 reads, every 1 min) at a wavelength of 490–630 nm in a ELx808 BioTek plate reader. The level of complement activation was expressed as percentage of lysis of target cells compared to maximum lysis included by the addition of 4% Triton-X100.

### NK cell degranulation and antibody-dependent cellular cytotoxicity

Degranulation of NK cells was assessed using a modified flow cytometry-based assay using surface mobilization of CD107a as a readout as described previously [52]. Pig peripheral blood mononuclear cells (PBMCs) were stimulated overnight with recombinant pig IL-2 (20 ng/ml; Kingfisher Biotech, Saint Paul, MN), IL-12 (25 ng/ml), and IL-18 (100 ng/ml; both from R&D Systems, Minneapolis, MN). ELISA plates (Nunc Maxisorp) were coated overnight at 4°C with 5 µg/well of recombinant HA against respective mAbs (HK5738 for H3, VN1203 for H5 and Anh1 for H7) in carbonate-bicarbonate buffer. After three washes, plates were blocked with 1% BSA in PBS for 1 h at room temperature (RT).

Monoclonal antibodies against H3, H5 and H7, were added to respective HA coated or uncoated wells and incubated for 1 h at RT. Stimulated PBMCs were washed, re-suspended in AIM-V medium, and 1 × 10^6^ cells were added per well. Anti-CD107a-FITC (clone 4E9/11; IgG1, Bio-Rad) at 4 µg/ml, brefeldin A (GolgiPlug, BD Biosciences), and monensin (GolgiStop, BD Biosciences) were added, and cells were incubated for 5 hours at 37°C. Following incubation, cells were transferred to U-bottom 96-well plate, washed and stained with a live/dead near-infrared viability dye (Thermo Fisher), anti-CD3ε (clone BB23-8E6-8C8, BD Biosciences), and anti-CD8α (clone 76-2-11, BD Biosciences) for 15 minutes at RT. After washing and fixation with 4% paraformaldehyde, samples were acquired using a MACSQuant 10 flow cytometer (Miltenyi Biotec). Data were exported and percentage of NK cell defined as CD3^−^CD8a^+^ expressing CD107a were analysed using FlowJo v10 software (BD Life Sciences).

## Supplementary Material

kyag006_Supplementary_Data

## Data Availability

Data generated or analysed during this study that are critical to the reported findings are available within the article and its Supplementary Information files. Monoclonal antibodies and sequence information generated in this study are available from the corresponding authors upon reasonable request, for non-commercial research use.

## Permission to reproduce

Graphical abstract created in BioRender. May, C. (2026) https://BioRender.com/a6dm9gm

## Abbreviations:

H or HA: haemagglutinin
HAI: haemagglutination inhibition
mAb: monoclonal antibodies
