# Supplementary material for: Porcine influenza mAbs to H3, H5, and H7 hemagglutinins recognize H3 egg adapted site and target the HA stem
Source: Discov Immunol. 2026 Mar 2;5(1):kyag006. doi: 10.1093/discim/kyag006 (PMC13006140; doi:10.1093/discim/kyag006)
Supplement: kyag006_Supplementary_Data [file kyag006_supplementary_data.zip › Supplementary Figures 7th october.pdf]

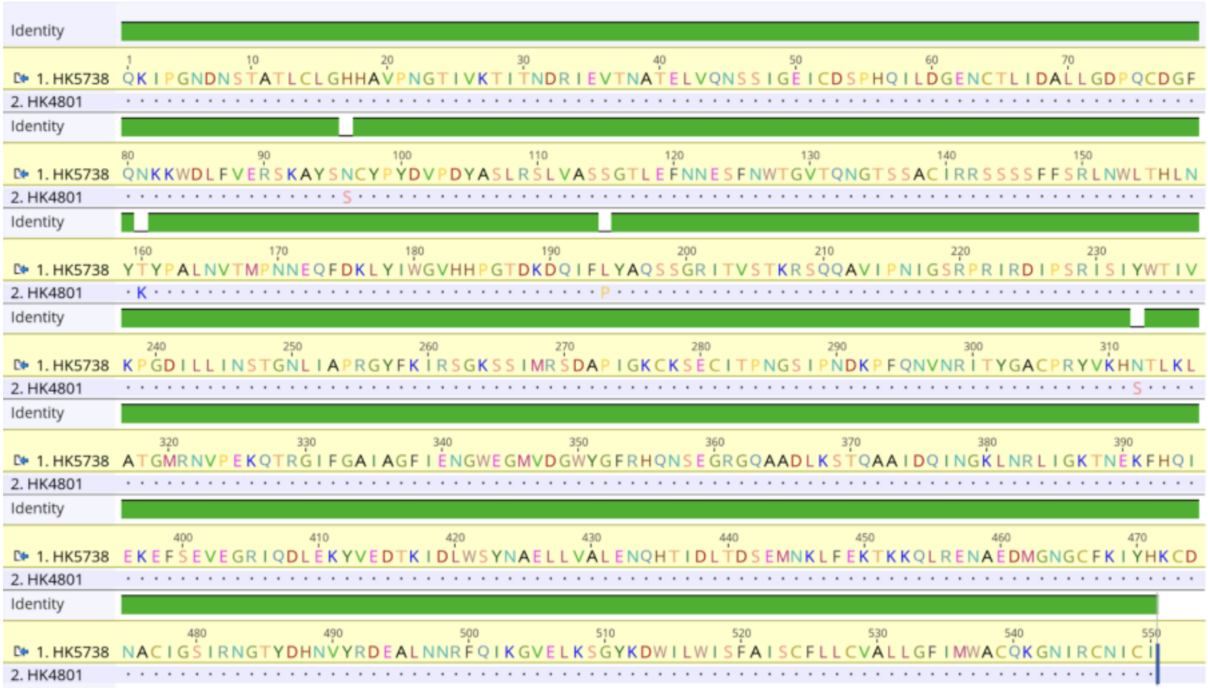

Supplementary Figure 1. Sequence alignment of H3N2 HA from the egg-grown vaccine strain HK4801 and the HA from HK5738 used for sorting. HK4801 virus shows egg-adaptative substitutions in antigenic site B (T160K and L194P).

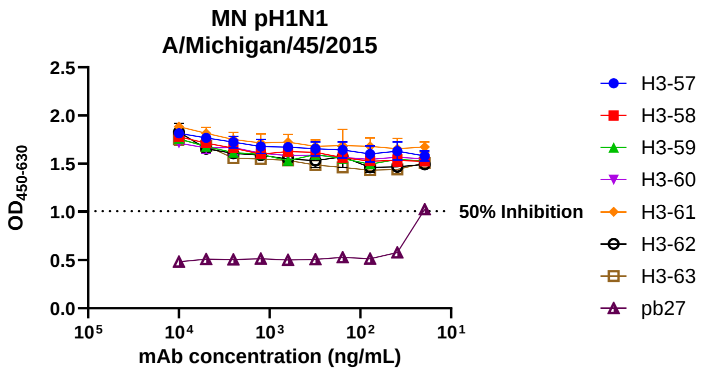

Supplementary Figure 2: H3 mAbs MN with H1N1pdm09 (A/Michigan/45/2015)

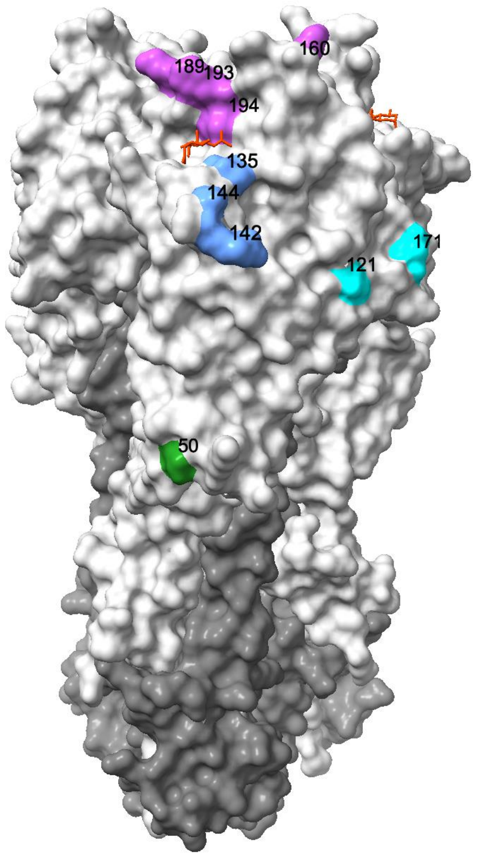

Supplementary Figure 3: Structure of H3 HA showing key antigenic sites. PDB 6BKT (Trimeric HA assembly of H3N2 HA A/Michigan/15/2014) was visualized using ChimeraX 1.10. HA1 domains are shown in light grey and HA2 domains in dark grey. Antigenic sites A-D are shown in distinct colors in a HA monomer: site A (residues 135, 142, 144) in blue; site B (160, 189, 193, 194) in purple; site C (50) in green; and site D (121, 171) in cyan. The sialic acid at the receptor-binding site is shown in orange.

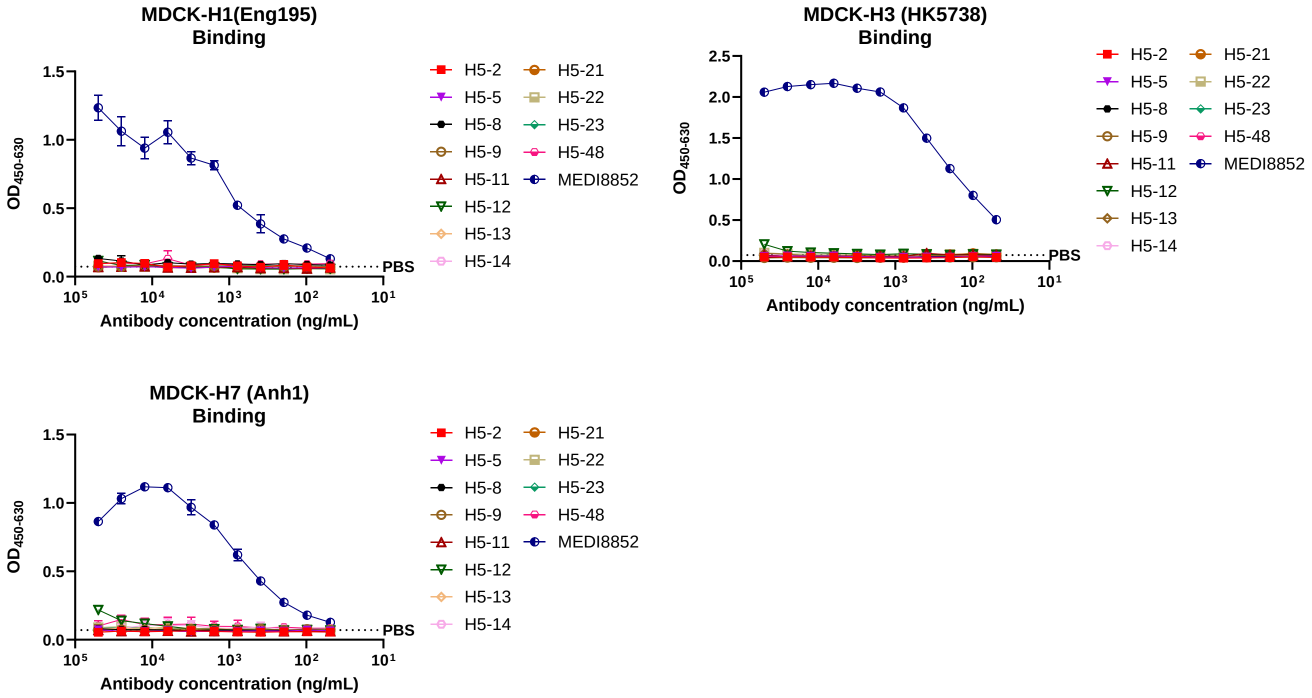

Supplementary Figure 4. Binding to MDCK cells. H5 mAbs binding with MDCK-H1 (Eng195), MDCK-H3 (HK5738) and MDCK-H7 (Anh1). Error bars represent the standard deviation.

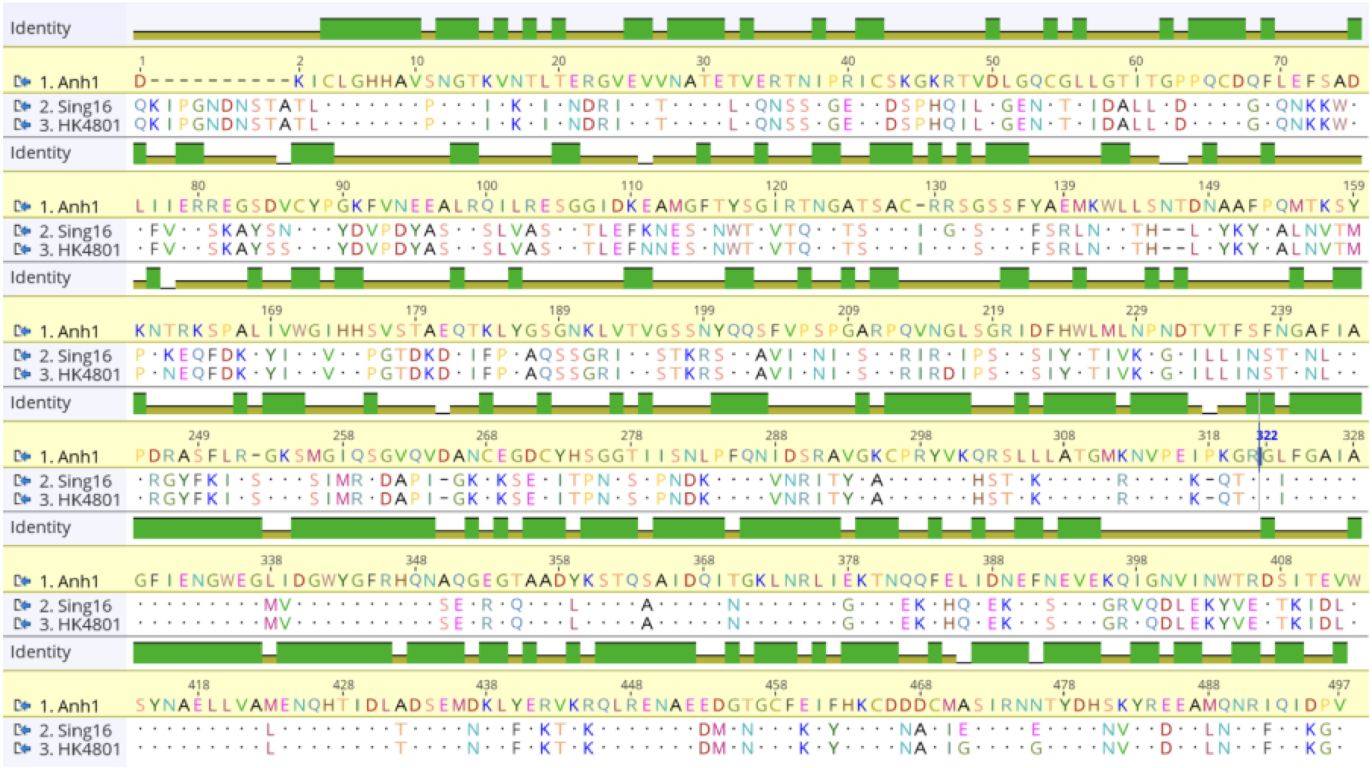

Supplementary Figure 5. Sequence conservation between H7 HA (Anh1) and H3 HA (Sing16, and HK4801). R329 (H3 numbering) marks the HA1/HA2 boundary. There is higher sequence similarity for HA2 than HA1. Alignment was done using Muscle alignment in Geneious Prime.
