# Supplementary material for: Porcine influenza mAbs to H3, H5, and H7 hemagglutinins recognize H3 egg adapted site and target the HA stem
Source: Discov Immunol. 2026 Mar 2;5(1):kyag006. doi: 10.1093/discim/kyag006 (PMC13006140; doi:10.1093/discim/kyag006)
Supplement: kyag006_Supplementary_Data [file kyag006_supplementary_data.zip › Supplementary Tables 31st Oct.pdf]

Supplementary Table 1: MN assay against the HK/2014 S-FLU virus and 11 single mutant variants

	WT	E50K	T160K	F193S	L194P	T135K	S144N	S144K	R142G	K189E	N121K	N171K
H3-59	neut	neut	neut	neut	+/-	neut	neut	neut	neut	neut	neut	neut
H3-60	neut	neut	neut	neut	neut	neut	neut	neut	neut	neut	neut	neut
H3-61	-	-	-	-	-	-	-	-	-	-	-	-
H3-62	neut	neut	neut	neut	neut	neut	neut	neut	neut	neut	neut	neut
H3-63	-	-	-	-	-	-	-	-	-	-	-	-
MEDI8852	neut	neut	neut	neut	neut	neut	neut	neut	neut	neut	neut	neut

PCR I:

Heavy chain (IgG)

Reagent	X1 (µl)
Nuclease-free Water	9.82
5x Q5 reaction buffer	5
5x Q5 GC enhancer	5
10 mM dNTPs	0.5
10 µM Primer Fw	0.25
10 µM Primer Rev	0.25
Q5 Hot Start High-Fidelity DNA polymerase (M0493L, NEB)	0.18
Template (cDNA)	4
Total	25

Heavy chain IgG thermocycler program

	Temp	Time
Initial denaturation	98°C	30 sec
25 cycles	98 °C	10 sec
	64 °C	20 sec
	72 °C	20 sec
Extension	72 °C	2 minutes

Light chain (Igk)

Reagents	X1 (µl)
Nuclease-free Water	9.57
5x Q5 reaction buffer	5
10 mM dNTPs	0.5
10 µM Primer Fw-1	0.25
10 µM Primer Fw -2	0.25
10 µM Primer Rev	0.25
5x Q5 GC Enhancer	5
Q5 Hot Start High-Fidelity DNA polymerase	0.18
Template (cDNA)	4
Total	25

Light chain kappa thermocycler program

	Temp	Time
Initial denaturation	98°C	30 sec
30 cycles	98 °C	10 sec
	64 °C	20 sec
	72 °C	20 sec
Extension	72 °C	2 minutes

Light chain lambda

Reagents	X1 (µl)
Nuclease-free Water	9.32
5x Q5 reaction buffer	5
5x Q5 GC Enhancer	5
10 mM dNTPs	0.5
10 µM Primer Fw-1	0.25
10 µM Primer Fw -2	0.25
10 µM Primer Fw -3	0.25
10 µM Primer Rev	0.25
Q5 Hot Start High-Fidelity DNA polymerase	0.18
Template (cDNA)	4
Total	25

Light chain lambda thermocycler program

	Temp	Time
Initial denaturation	98°C	30 sec
30 cycles	98 °C	10 sec
	64 °C	20 sec
	72 °C	20 sec
Extension	72 °C	2 minutes

PCR III (Overhang addition):

Heavy chain (IgG)

Reagents	X1 (µl)
Nuclease-free Water	12.57
5x Q5 reaction buffer	5
10 mM dNTPs	0.5
10 µM Primer Fw	0.25
10 µM Primer Rev -1	0.25
10 µM Primer Rev-2	0.25
5x Q5 GC Enhancer	5
Q5 Hot Start High-Fidelity DNA polymerase	0.18
IgG PCR I product diluted (1:10) or 1:20 PCR II product.	1
Total	25

Heavy chain thermocycler program

	Temp	Time
Initial denaturation	98°C	30 sec
5 cycles	98 °C	10 sec
	64 °C	20 sec
	72 °C	15 sec
25 cycles	98 °C	10 sec
	68 °C	20 sec
	72 °C	15 sec
Extension	72 °C	2 minutes

Light chain kappa

Reagents	X1 (µl)
Nuclease-free Water	12.57
5x Q5 reaction buffer	5
10 mM dNTPs	0.5
10 µM Primer Fw-1	0.25
10 µM Primer Fw -2	0.25
10 µPrimer Rev	0.25
5x Q5 GC Enhancer	5
Q5 Hot Start High-Fidelity DNA polymerase	0.18
IgK PCR I template (diluted 1:10)	1
Total	25

Light chain kappa thermocycler program

	Temp	Time
Initial denaturation	98°C	30 sec
5 cycles	98 °C	10 sec
	65 °C	20 sec
	72 °C	15 sec
25 cycles	98 °C	10 sec
	68 °C	20 sec
	72 °C	15 sec
Extension	72 °C	2 minutes

Light chain lambda

Reagents	X1 (µl)
Nuclease-free Water	12.32
5x Q5 reaction buffer	5
10 mM dNTPs	0.5
10 µM Primer Fw-1	0.25
10 µM Primer Fw -2	0.25
10 µM Primer Fw -3	0.25
10 µM Primer Rev	0.25
5x Q5 GC Enhancer	5
Q5 Hot Start High-Fidelity DNA polymerase	0.18
IgL PCR I template (diluted 1:10)	1
Total	25

Light chain lambda thermocycler program

	Temp	Time
Initial denaturation	98°C	30 sec
5 cycles	98 °C	10 sec
	65 °C	20 sec
	72 °C	15 sec
25 cycles	98 °C	10 sec
	68 °C	20 sec
	72 °C	15 sec
Extension	72 °C	2 minutes

PCRIV (Indexing):

Reagents	X1 (µl)
Nuclease-free Water	6.85
5x Q5 reaction buffer	5
10 mM dNTPs	0.5
1 µM Primer Fw	2.5
1 µM Primer Rev	2.5
5x Q5 GC Enhancer	5
Q5 Hot Start High-Fidelity DNA polymerase	0.15
PCR III purified product*	2.5
Total	25

Indexing thermocycler program

	Temp	Time
Initial denaturation	98°C	30 sec
10 cycles	98 °C	10 sec
	58°C	20 sec
	72 °C	20 sec
Extension	72 °C	2 minutes

Supplementary Table 2: PCR amplification, indexing of IgG heavy and light chain.
